# Targeting MDM2, RAS, and PCNA for cancer targeted therapy: pan-cancer approaches vs. cancer-specific strategies

**DOI:** 10.3389/fphar.2025.1663766

**Published:** 2025-10-15

**Authors:** Wei Wang, Abigail Alley, Na Sun, Meheret Tadesse, Xinshi Wang, Ruiwen Zhang

**Affiliations:** ^1^ Department of Pharmacological and Pharmaceutical Sciences, College of Pharmacy, University of Houston, Houston, TX, United States; ^2^ Drug Discovery Institute, University of Houston, Houston, TX, United States

**Keywords:** MDM2, Ras, PCNA, pan-cancer, cancer-specific

## Abstract

Cancer therapy and cancer drug discovery and development have been historically focused on specific cancers (tissue/organ of origin). However, with advances in molecular biology and multi-omics of cancer, there is a trend to develop pan-cancer therapeutic modalities. In targeted therapy, pan-cancer strategies target common molecular alterations across different cancer types and specific cancer strategies are tailored to the unique biological characteristics of individual tumor types. Each approach offers distinct advantages and limitations, and understanding these differences is critical in the era of precision oncology. Targeting key molecular drivers in cancer has significantly changed drug development, allowing for broad-spectrum therapeutic strategies that address shared oncogenic pathways across various tumor types. Among these drivers, RAS, PCNA, and MDM2 have become critical targets due to their roles in a broad-spectrum of cancer biology, e.g., cell proliferation, survival, and genomic stability. Advances in molecularly guided therapies have led to promising approaches for disrupting these pathways, offering new opportunities for cancer treatment. Despite significant progress in the past, challenges such as drug resistance, tumor heterogeneity, and toxicity remain obstacles to widespread clinical success. This review explores the historical development, current advancements, and future directions of RAS, PCNA, and MDM2-targeted therapies, emphasizing their potential to reshape cancer treatment through pan-cancer approaches using biomarker-driven technologies, combination strategies, and next-generation inhibitors. These advancements pave the way for more effective and durable therapies across a wide range of malignancies.

## 1 Introduction

The landscape of cancer therapy has evolved dramatically over the past few decades, transitioning from traditional chemotherapy and radiotherapy to more precise, molecularly targeted treatments, including advanced immunotherapy (Liu et al., 2024). Two major paradigms have emerged in this evolution of cancer treatment modalities: pan-cancer strategies, which target common molecular alterations across different cancer types, and specific cancer strategies, which are tailored to the unique biological characteristics of individual tumor types. Each approach offers distinct advantages and limitations, and understanding these differences is critical in the era of precision oncology.

### 1.1 Specific cancer strategies

Historically, basic oncological research and clinical oncology practice are often based on specific cancer therapy strategies, tailoring treatments to the unique characteristics of a particular cancer type or organ site (Liu et al., 2024; Liu et al., 2021). This approach leverages detailed histological and cellular/molecular profiling to identify targets that are uniquely relevant to a given tumor. For instance, the major regimens are developed for specific cancers according to clinical staging. In respect of targeted therapy, the overexpression of the HER2 receptor in certain breast cancers has led to the development of targeted therapies such as trastuzumab (Slamon et al., 2011), which have dramatically improved patient outcomes in this subset of patients. The primary strength of cancer-specific strategies lies in their high degree of precision. By considering the unique genetic, molecular, and environmental factors associated with a specific cancer type, these therapies can be optimized to maximize efficacy while minimizing off-target effects. Detailed profiling allows clinicians to select the most appropriate treatment for each patient, while at the same time aligning with the principles of personalized medicine. Cancer-specific approaches also facilitate the development of combination therapies tailored to the complex molecular networks of individual tumors (Boshuizen and Peeper, 2020). For example, in cancers driven by multiple concurrent mutations or aberrations, combining agents that target different pathways may provide a synergistic effect, overcoming resistance mechanisms that often limit the efficacy of monotherapies (Boshuizen and Peeper, 2020). Despite these advantages, cancer-specific strategies also face significant challenges. One of the primary obstacles is the complexity and cost associated with developing therapies for narrowly defined patient populations. Each cancer type may require its own set of clinical trials, which can be both time-consuming and resource-intensive. Furthermore, the results from a trial in one specific cancer may not be easily generalizable to other cancers, even if they share similar molecular features. This fragmentation of research efforts can slow the pace of innovation and limit the broader applicability of new treatments. Additionally, while high specificity is beneficial for targeted therapy, it can also limit treatment options for patients whose tumors do not harbor well-defined or actionable molecular targets (Zhang et al., 2020). In such cases, the lack of a clear target may necessitate reliance on broader-spectrum therapies, which may not provide the same level of efficacy or reduced toxicity associated with more precise interventions.

### 1.2 Pan-cancer strategies

Pan-cancer approaches are founded on the concept that certain molecular alterations drive cancer development regardless of the tissue of origin (Campanharo et al., 2024). These strategies aim to exploit common pathways or biomarkers present in multiple cancer types. For instance, mutations in genes involved in cell cycle regulation or signaling pathways, such as alterations in the RAS pathways, can be found across a variety of cancers (Chen et al., 2021; Singh et al., 2023). One notable advantage of pan-cancer strategies is their broad applicability. By targeting these shared molecular aberrations, pan-cancer therapies offer the potential to treat a diverse patient population with a single therapeutic agent (Duan et al., 2020). With advances in genomic profiling, researchers have identified recurrent genetic alterations that occur in a significant subset of cancers, irrespective of the tumor’s anatomical origin (Campanharo et al., 2024). Tissue-agnostic therapies, such as those targeting microsatellite instability-high (MSI-H) tumors (Yamamoto et al., 2024) or neurotrophic receptor tyrosine kinase (NTRK) gene fusions (Theik et al., 2024), have already entered clinical practice, demonstrating the promise of this approach. The FDA approval of pembrolizumab for MSI-H tumors is a prime example of how a pan-cancer strategy can lead to effective treatment across multiple cancer types (Cindy Yang et al., 2021). Additionally, pan-cancer trials often benefit from more streamlined clinical designs. By focusing on a specific molecular target rather than the tumor type, these trials can enroll patients based on the presence of a biomarker rather than the traditional classification by tissue. This strategy not only accelerates the recruitment process but also potentially reduces the time needed to bring a new drug to market. However, the pan-cancer approach is not without its challenges. One significant limitation is the heterogeneity of tumor biology (Proietto et al., 2023; Zhu et al., 2021). Even when tumors share a common mutation or molecular pathway, the context in which these alterations occur can vary significantly between different cancer types (Proietto et al., 2023; Zhu et al., 2021). For example, the microenvironment, co-existing genetic mutations, and epigenetic factors can influence how a tumor responds to a targeted therapy. As a result, a drug that is highly effective in one type of cancer may be less so in another, despite both harboring the same molecular target. Furthermore, the complexity of cancer biology means that a single molecular target may not fully capture the intricacies of tumor behavior. While targeting a common pathway can be beneficial, it may also lead to oversimplification, ignoring other critical factors that contribute to tumor growth and resistance. This limitation underscores the importance of ongoing research to understand the full spectrum of molecular interactions within tumors.

### 1.3 Targeting driver oncogenes

Despite remarkable advancements in cancer therapies, addressing the molecular underpinnings of tumorigenesis remains a cornerstone of modern oncology. Cancer is a complex and multifaceted disease driven by the dysregulation of critical molecular pathways that regulate fundamental cellular processes, including growth, proliferation, apoptosis, and genomic stability (Hanahan, 2022). Among the diverse hallmarks of cancer, a subset of critical molecular drivers, proteins, and signaling pathways consistently dysregulated across various tumor types, has emerged as compelling therapeutic targets (Hanahan, 2022; Liu et al., 2024). These drivers offer opportunities for broad-spectrum treatments, providing a unified approach to managing malignancies with diverse origins and characteristics (Liu et al., 2024).

Focusing on molecular targets is crucial to overcoming the complexity and heterogeneity of cancer. Key molecules such as proliferating cell nuclear antigen (PCNA), rat sarcoma virus (RAS) proteins, and mouse double minute 2 (MDM2) are central to essential biological processes like DNA replication, repair, proliferation, and apoptosis (Wang and Wang, 2025; Wang W. et al., 2024; Yang and Wu, 2024). Dysregulation of these processes often underpins cancer progression. For example, PCNA, a key regulator of DNA replication and repair, is frequently overexpressed or modified in tumors, contributing to their growth and survival (Wang and Wang, 2025). RAS proteins, which act as molecular switches controlling multiple signaling pathways, are among the most frequently mutated oncogenes in cancer, leading to uncontrolled cell division and resistance to apoptosis (Yang and Wu, 2024). Similarly, MDM2, a negative regulator of the tumor suppressor p53, is often amplified in cancers, enabling tumor cells to evade cell death and resist therapy (Wang W. et al., 2024). The identification of these molecular drivers has transformed cancer drug development by enabling precise intervention in core cellular pathways essential for tumor growth and survival.

Recent advancements in targeting key molecular drivers have showcased the potential for innovative therapies that can be applied across a wide range of cancer types. A prime example is the development of novel PCNA inhibitors, which aim to disrupt DNA replication and repair, the key processes essential for tumor proliferation. Among these, AOH1996, a first-in-class small-molecule PCNA inhibitor, selectively targets cancer-associated PCNA isoforms, effectively impairing DNA replication and repair in tumor cells while sparing normal cells (Gu et al., 2023b). Similarly, targeting RAS mutations, particularly the KRAS G12C variant, one of the most frequent oncogenic drivers, has long been considered “undruggable” due to the lack of suitable drug-binding pockets. However, groundbreaking progress has been made with the FDA approval of KRAS G12C inhibitors such as Sotorasib (AMG510) (Skoulidis et al., 2021) and Adagrasib (MRTX849) (Jänne et al., 2022), marking a significant milestone in cancer therapy. In parallel, significant strides have been made in the development of second-generation MDM2 inhibitors, which exhibit improved potency and reduced toxicity. While no MDM2-targeting drug has yet received FDA approval, a promising new strategy involves targeting MDM2 degradation is currently under investigation (Wang W. et al., 2024). This approach has the potential to be effective regardless of the p53 status of cancer, opening new avenues for broader therapeutic applications. Notably, the FDA recently granted orphan drug designation to KT-253, a novel MDM2 degrader, for the treatment of acute myeloid leukemia (AML), underscoring the clinical potential of this innovative strategy (Wang W. et al., 2024). These advances highlight the promise of molecularly guided therapies, which not only provide precision-based treatment strategies but also broaden therapeutic possibilities across multiple cancer types, reshaping the oncology landscape.

The ability to target shared molecular vulnerabilities across diverse cancers offers an opportunity to streamline drug development, reducing the need for entirely new drugs for each cancer subtype. In this review, we will examine PCNA, RAS, and MDM2 as key examples, exploring their roles in tumorigenesis, the challenges associated with targeting these molecular drivers, and the breakthroughs that have enabled the development of inhibitors aimed at disrupting their oncogenic functions. By assessing the current landscape of drug development for these targets, this review highlights their significance as critical regulators of cancer progression and emphasizes the potential of innovative therapies to drive transformative advances in oncology, paving the way for more effective, broad-spectrum cancer treatments. These oncogenes have been well investigated, and their inhibitors have been discovered and developed, some of which have been approved for clinical use and entered clinical trials. Interested readers are directed to several recent, excellent publications (Ash et al., 2024; Cardano et al., 2020; Cox and Der, 2025; D’Alessio-Sands et al., 2025; Horsfall et al., 2020; Isermann et al., 2025; Li et al., 2024; Linette et al., 2024; Molina-Arcas and Downward, 2024; Pandey et al., 2024; Perurena et al., 2024; Singhal et al., 2024; Søgaard and Otterlei, 2024; Sun et al., 2024; Twarda-Clapa, 2024; Wang and Wang, 2025; Wang W. et al., 2024; Wang et al., 2025; Wendel et al., 2023; Wu et al., 2023; Yao et al., 2024; Zafar et al., 2021; Zeng et al., 2024). Although we will emphasize the aspects of pan-cancer approaches in the review, a comprehensive understanding of biology and the discovery and development of therapeutics targeting those genes will be helpful in exploring specific cancer targeting strategies.

## 2 Targeting the RAS pathways: a 50-year learning curve

### 2.1 RAS, a driver gene in cancer

RAS proteins are among the most extensively studied molecular regulators in cellular biology, playing a critical role in regulating essential processes such as cell proliferation, differentiation, and survival (Yang and Wu, 2024). Encoded by the RAS gene family, these small GTPases serve as key mediators of intracellular signaling pathways that translate extracellular signals into cellular responses (Yang and Wu, 2024). Mutations in RAS, particularly in its most frequently altered isoform, KRAS, are strongly associated with a variety of human cancers, establishing RAS as one of the most commonly mutated oncogenes (Burge and Hobbs, 2022). Since its discovery in the 1960s (Harvey, 1964), RAS has become a cornerstone of cancer biology, with its intricate structure, biological functions, and role in tumorigenesis offering profound insights into cancer development and progression. Despite decades of extensive research, the complex roles of RAS in cellular signaling and its multifaceted implications in cancer biology remain a focal point of cutting-edge scientific inquiry (Punekar et al., 2022). Recent breakthroughs, such as the FDA approval of two RAS inhibitors, Adagrasib (Krazati) and Sotorasib (Lumakras), represent significant milestones in targeting this historically elusive oncogene (Jänne et al., 2022; Skoulidis et al., 2021; Wu et al., 2023). These advancements highlight progress in overcoming challenges in RAS-directed therapies, underscoring its critical importance as a central target in molecular oncology. This enduring focus drives ongoing efforts to unravel RAS’s intricate mechanisms and develop innovative strategies to combat RAS-driven cancers.

The RAS family of proteins, encoded by the *RAS* oncogene, are small GTP-binding proteins that play a central role in regulating cellular processes such as differentiation, proliferation, and survival. These proteins exist in four main subtypes: KRAS4A, KRAS4B, NRAS, and HRAS. RAS proteins function as molecular switches, cycling between an active GTP-bound state (RAS-GTP) and an inactive GDP-bound state (RAS-GDP). This cycling is tightly regulated by two key classes of proteins: Ras-GTPase activating proteins (GAPs), which promote GTP hydrolysis to inactivate RAS, and guanine nucleotide exchange factors (GEFs), which facilitate the exchange of GDP for GTP to activate RAS (Gasper and Wittinghofer, 2019). This precise regulation ensures that RAS signaling is tightly controlled in response to cellular functions.

Mutations in RAS lock it in an active GTP-bound state, leading to dysregulated signaling through multiple downstream effectors, including rapidly accelerated fibrosarcoma (RAF) kinases, phosphoinositide 3-kinases (PI3K), the RAS association domain family (RASSF), T lymphoma invasion and metastasis protein 1 (TIAM1), Ral guanine nucleotide dissociation stimulator (RALGDS), phospholipase Cε (PLCε), novel RAS effector 1A (NORE1A), Af6, RAS and Rab interactor 1 (RIN1), growth factor receptor 14 (Grb14), and the lysine methyltransferase (KMT2A)-polo-like kinase 1 (PLK1) axis (Carr et al., 2021; Jagadeeshan et al., 2023; Stephen et al., 2014). This aberrant signaling drives tumorigenic processes, including uncontrolled cell proliferation, differentiation, and evasion of apoptosis.

Among RAS effector pathways, the RAF-mitogen-activated protein kinase kinase-extracellular signal-regulated kinase (RAF-MEK-ERK) cascade is the most well-characterized. In its GTP-bound active state, RAS recruits RAF kinases to the plasma membrane, triggering their activation. Once activated, RAF phosphorylates MEK1/2, which subsequently phosphorylates and activates ERK1/2 (Ullah et al., 2022). Activated ERK translocates to the nucleus, where it regulates the transcription of genes involved in oncogenic signaling (Ullah et al., 2022). Structural studies have provided key insights into RAF activation by RAS, revealing that RAS dimerization is crucial for effective RAF activation, and disruption of RAS dimers has been proposed as a potential therapeutic strategy (Herrero and Crespo, 2021). Additionally, cryo-EM structural analysis of the RAS-RAF complex has demonstrated that RAS binding alone is insufficient to activate RAF, highlighting the need for additional regulatory interactions (Park et al., 2023). Beyond the RAF-MEK-ERK cascade, the PI3K-AKT pathway serves as another major RAS effector, governing cell survival, metabolism, and growth. RAS activates PI3K, leading to the production of phosphatidylinositol (3,4,5)-trisphosphate (PIP3), a lipid second messenger that recruits AKT to the plasma membrane for activation (Castellano and Downward, 2011; Cuesta et al., 2021). Activated AKT phosphorylates multiple downstream targets, regulating apoptosis, metabolism, and other essential cellular processes (Castellano and Downward, 2011; Cuesta et al., 2021).

**FIGURE 1:**
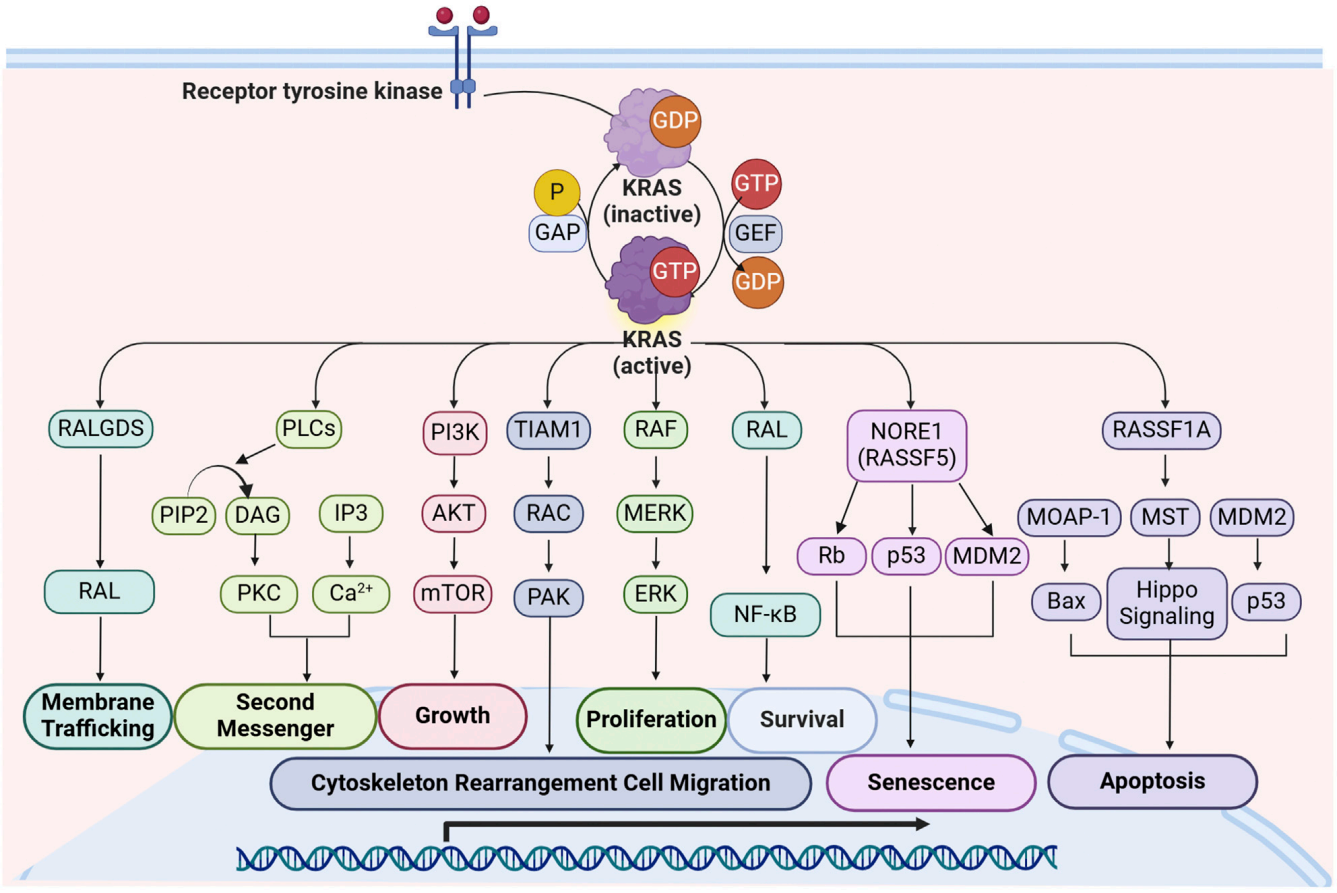
Overview of KRAS signaling and downstream effector pathways. Upon activation by RTKs, KRAS interacts with multiple effectors, initiating critical cellular processes such as proliferation, growth, apoptosis, migration, and survival. The major signaling cascades include: RALGDS Pathway: KRAS activates RALGDS, leading to RAL activation, which facilitates membrane trafficking and vesicular transport; PLC Pathway: KRAS engages PLC, which hydrolyzes PIP2 into DAG and IP3. This promotes PKC activation and Ca^2+^ release, contributing to second messenger signaling; PI3K-AKT-mTOR Pathway: KRAS activates PI3K, leading to the phosphorylation of AKT and subsequent activation of mTOR, promoting cell growth and metabolic regulation; TIAM1-RAC-PAK Pathway: KRAS activation of TIAM1 leads to RAC and PAK activation, which regulate cytoskeletal rearrangement and cell migration, contributing to tumor metastasis; RAF-MEK-ERK Pathway: KRAS recruits RAF kinases, initiating a phosphorylation cascade involving MEK and ERK, which drives cell proliferation by regulating transcription factors and gene expression; RAL-NF-κB Pathway: KRAS activates RAL, which in turn activates NF-κB, supporting cell survival and resistance to apoptosis; NORE1A (RASSF5) Pathway: KRAS interaction with NORE1A leads to activation of Rb and p53, promoting cellular senescence with regulation by MDM2; RASSF1A-MST Pathway: KRAS interacts with RASSF1A, which activates MST and the Hippo signaling pathway, leading to the activation of Bax and p53, promoting apoptosis and maintaining cellular homeostasis. Abbreviations: DAG: diacylglycerol; IP3: inositol triphosphate; MDM2: mouse double minute 2 Homolog; MOAP-1: modulator of apoptosis 1; MST: mercaptopyruvate sulfurtransferase; NORE1A: novel Ras effector 1A; PAK: p21-activated kinase; PIP2: Phosphatidylinositol 4,5-bisphosphate; PKC: protein kinase C; P LCε: phospholipase Cε; RALGDS: Ral guanine nucleotide dissociation stimulator; RASSF1A: RAS association domain family1 Isoform A; Rb: Retinoblastoma Protein; RTKs: receptor tyrosine kinases; TIAM1: T lymphoma invasion and metastasis protein 1.

RAS signaling is further diversified by its interactions with additional effectors, contributing to various cellular functions (Figure 1). A key factor influencing RAS signal specificity is its subcellular localization (Zhou and Hancock, 2021). RAS proteins are anchored to the plasma membrane through post-translational lipid modifications, forming nanoclusters that serve as highly efficient signaling hubs. These RAS nanoclusters enhance signaling specificity and pathway crosstalk (Zhou and Hancock, 2021). Disrupting RAS-membrane interactions has emerged as a novel strategy for inhibiting RAS-driven oncogenesis, providing a potential avenue for therapeutic intervention.

RAS mutations play a crucial role in cancer initiation and progression, making them one of the most significant oncogenic drivers across various cancer types. Meta-analyses have reported that approximately 19% of all cancer patients harbor RAS mutations, with KRAS mutations accounting for 75% of these cases (Prior et al., 2020). Due to their prevalence, KRAS-targeted therapies have been the primary focus of drug development efforts. However, all RAS isoforms (KRAS, NRAS, and HRAS) exhibit differential expression patterns in adult tissues and tumors, leading to distinct biological effects and therapeutic challenges. The frequency and distribution of RAS mutations vary among different types of cancer. KRAS mutations are predominantly found in pancreatic adenocarcinoma, colon adenocarcinoma, and rectal adenocarcinoma, whereas NRAS mutations are more common in skin cutaneous melanoma, anaplastic thyroid carcinoma, and follicular thyroid carcinoma (Prior et al., 2020). Given this widespread impact, pan-RAS inhibitors that target multiple RAS isoforms simultaneously have emerged as a promising strategy to overcome RAS-driven malignancies.

Analysis of PanCancer data from cBioPortal (Table 1) confirmed that KRAS (21%) was the most frequently mutated RAS isoform, primarily due to missense mutations, followed by NRAS (2%) (by Sep, 2025). The most commonly mutated residues in RAS-driven cancers included Gly12, Gln61, and Gly13, with KRAS mutations at codon 12 being particularly dominant in pancreatic adenocarcinomas (Smit et al., 1988) and other exocrine pancreatic carcinomas (Almoguera et al., 1988). However, not all RAS mutations are equal, as their functional impact varies by cancer type, isoform, and mutation site (Burge and Hobbs, 2022). In thyroid carcinoma, a systematic review and network meta-analysis confirmed that RAS mutations negatively impact long-term prognosis (Zhao et al., 2020). Similarly, the *HRAS* G12S mutation has been linked to poor prognosis in head and neck squamous cell carcinoma, as it enhances angiogenesis and reduces responsiveness to chemotherapy (Sambath et al., 2024). In melanomas, NRAS mutations are the second most common alteration, found in approximately 25% of cases, second only to BRAF mutations (40%–45%) (Randic et al., 2021). By contrast, KRAS and HRAS mutations are far less frequent in melanomas, occurring in about 5% of cases (Randic et al., 2021). Unlike KRAS, NRAS, and HRAS, MRAS mutations are rare in human cancers due to their lower affinity for RAF proteins and reduced ability to activate the ERK pathway (Endo, 2020). However, cBioPortal data (by September 2025) suggest that MRAS amplification or overexpression occurs at notable frequencies in esophageal squamous cell carcinoma (10.53%), lung squamous cell carcinoma (9.03%), cervical carcinoma (8.37%), uterine serous carcinoma (6.42%), ovarian carcinoma (5.48%), and head and neck squamous cell carcinoma (4.97%), indicating a potential role in tumor progression.

**TABLE 1:** Frequency of RAS isoform mutations in Top 20 Cancer Types.

Cancer types	Total rate (%)	RAS mutations (%)				
		K-ras	H-ras	N-ras	M-ras	R-ras
Pancreatic Cancer	74.45	73.82	0.06	0.48	0.00	0.09
Appendiceal Cancer	60.39	59.11	0.00	1.28	0.00	0.00
Small Bowel Carcinoma	59.64	57.89	0.00	1.75	0.00	0.00
Colorectal Carcinoma	51.84	47.76	0.73	3.35	0.00	0.00
Small Bowel Cancer	46.63	46.00	0.00	0.00	0.00	0.63
Colorectal Cancer	45.82	40.97	0.68	3.95	0.22	0.00
Extrahepatic Cholangiocarcinoma	35.40	33.05	0.00	1.71	0.00	0.64
Intrahepatic Cholangiocarcinoma	29.55	27.03	0.00	2.52	0.00	0.00
Non-Small Cell Lung Cancer	26.67	25.43	0.28	0.87	0.09	0.00
Ampullary Cancer	24.62	24.24	0.00	0.00	0.00	0.38
Uterine Corpus Endometrial Carcinoma	24.59	19.67	1.64	3.28	0.00	0.00
Endometrial Cancer	19.42	15.76	0.69	2.62	0.00	0.35
Lung Cancer	16.37	12.73	3.64	0.00	0.00	0.00
Uterine Endometrioid Carcinoma	16.67	12.50	0.00	4.17	0.00	0.00
Cancer of Unknown Primary	14.97	12.16	0.15	2.43	0.00	0.23
Gastrointestinal Neuroendocrine Tumor	13.86	11.55	0.00	2.31	0.00	0.00
Ovarian Carcinoma	11.97	9.96	0.77	1.24	0.00	0.00
Germ Cell Tumor	11.54	8.92	0.00	2.62	0.00	0.00
Non Small Cell Lung Cancer	11.25	10.00	0.28	0.59	0.00	0.38
lbladder Carcinoma	10.00	9.17	0.00	0.83	0.00	0.00

Data sources from cBioPortal.org (date to 11 Sep 2025). Total cases were more than 10 and Ras isoform gene altered frequencies above 10% are listed.

Importantly, RAS mutations rarely occur in isolation. A large-scale genomic study analyzing >600,000 alterations from >66,000 cancer patients across 51 tumor types revealed that RAS-mutant tumors exhibit context-dependent genomic profiles, often co-occurring with other oncogenic mutations (Scharpf et al., 2022). These findings support combination strategies, integrating RAS-targeted therapies with other targeted agents and immunotherapy to enhance clinical outcomes. Understanding the genomic diversity of RAS-driven cancers will be crucial in refining personalized treatment approaches and overcoming drug resistance mechanisms.

### 2.2 Targeting KRAS: challenges, failures, and breakthroughs

RAS has been recognized as a potential target for cancer therapy for nearly 4 decades (Chen et al., 2021). However, RAS proteins, particularly KRAS, were long considered “undruggable” due to a series of early failures in drug discovery (Cox and Der, 2025; Wu et al., 2023). Papke and Der also reviewed these setbacks, which included misconceptions about the function of mutant RAS proteins, leading to the failure of farnesyltransferase inhibitors (Papke and Der, 2017). Other challenges included the difficulty of competing with the strong binding affinity between RAS and the abundant cytoplasmic GTP, as well as the lack of suitable drug-binding pockets on the smooth surface of the RAS protein (Papke and Der, 2017).

**FIGURE 2:**
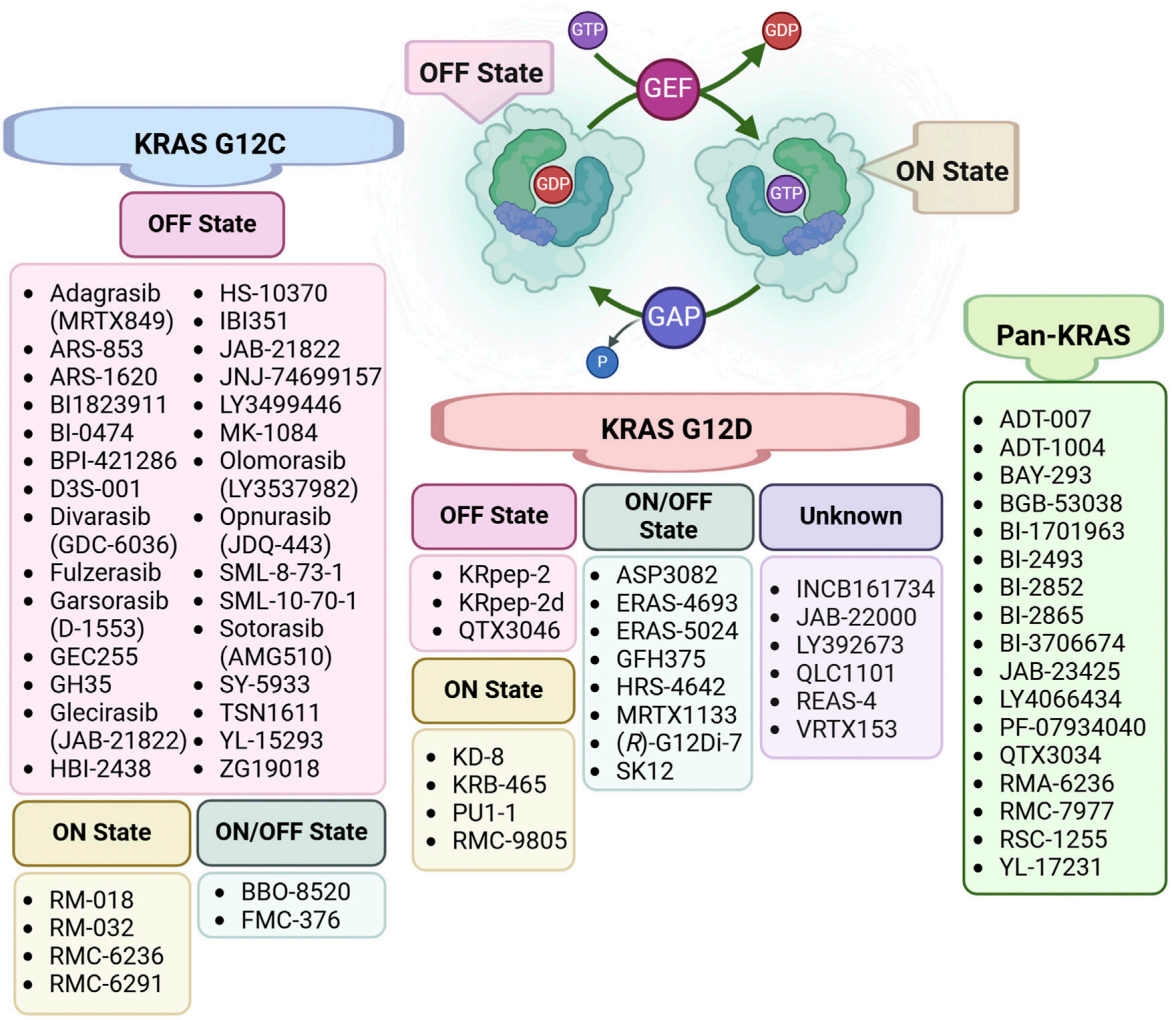
Classification of KRAS Inhibitors by Specific Targeting. The KRAS activation cycle is regulated by two key protein classes: GTPase-activating proteins (GAPs) and guanine nucleotide exchange factors (GEFs). GAPs facilitate the hydrolysis of GTP to GDP, driving KRAS into its inactive (OFF) state, while GEFs promote the exchange of GDP for GTP, shifting KRAS to its active (ON) state and enabling downstream signaling. This figure categorizes inhibitors based on their state-specific targeting of KRAS mutations, including KRAS G12C, KRAS G12D, and Pan-KRAS inhibitors.

The perception of KRAS as an “undruggable” target began to shift in 2013, when Ostrem et al. reported the first KRAS inhibitors that irreversibly bound to a new pocket beneath the switch II region, specifically targeting the cysteine residue in KRAS G12C (Ostrem et al., 2013). These inhibitors selectively affected the mutant protein without impacting the wild-type RAS, proving that KRAS could indeed be targeted therapeutically (Ostrem et al., 2013). This breakthrough marked the beginning of a new era in KRAS drug discovery, leading to rapid advancements over the following decade. As shown in Figure 2, the majority of currently developed KRAS inhibitors target the G12C mutation, which is one of the most common oncogenic drivers (Perurena et al., 2024; Singhal et al., 2024). A landmark achievement in targeting previously “undruggable” oncogenes was reached with the regulatory approval of the first KRAS G12C inhibitors. Sotorasib (AMG510) gained initial FDA authorization in 2021 (Skoulidis et al., 2021), and Adagrasib (MRTX849) was approved the following year (Jänne et al., 2022). Both drugs have shown promising efficacy in treating non-small cell lung cancer (NSCLC) and are structural derivatives of ARS-1620, a pioneering covalent inhibitor that selectively binds to the mutated cysteine in KRAS G12C (Jänne et al., 2022; Skoulidis et al., 2021). Building on this success, several other KRAS G12C inhibitors, such as Garsorasib (D-1553), Glecirasib (JAB-21822), and FMC-376, are currently undergoing clinical trials (Perurena et al., 2024; Shang et al., 2024; Singhal et al., 2024).

Despite these advancements, targeting other KRAS mutations, such as G12A, G12D, G12S, and G12R, remains in the early stages of development (Shang et al., 2024). For instance, RMC8839, the first oral inhibitor targeting KRAS^G13C^ (Escher and Satchell, 2023), and RM-046, a mutant-selective inhibitor of KRAS^Q61H^ (Yang YC. et al., 2023), are currently in preclinical studies. Beyond allele-specific inhibitors, complementary strategies such as targeted protein degradation, gene therapy, and cytosol-penetrating antibodies are being explored. Indirect targeting approaches, including inhibition of RAS nucleotide exchange factors (e.g., son of sevenless homolog 1 (SOS), src-homology 2 domain-containing phosphatase 2 (SHP2)), upstream regulators (e.g., epidermal growth factor receptor (EGFR)), and downstream signaling pathways (e.g., mitogen-activated protein kinase (MAPK), PI3K), also represent significant breakthroughs in the field (Yang H. et al., 2023).

In parallel, targeting the post-translational modifications (PTMs) of KRAS has been pursued as a therapeutic strategy to block its maturation and oncogenic signaling. Farnesyltransferase inhibitors (FTIs) such as L-744,832, lonafarnib, and tipifarnib were among the earliest agents developed to interfere with KRAS prenylation (Alcock et al., 2002; Basso et al., 2006; Karnoub and Weinberg, 2008; Kato et al., 1992; Marín-Ramos et al., 2019). While preclinical results were encouraging, clinical outcomes were disappointing because KRAS4B can bypass FTase inhibition through alternative geranylgeranylation by geranylgeranyltransferase 1 (GGTase-1) (Whyte et al., 1997). To overcome this resistance, dual FTase/GGTase-1 inhibitors such as L-778,123 were tested, but their limited ability to block KRAS prenylation and broad substrate toxicity constrained clinical application (Lobell et al., 2002). A next-generation compound, FGTI-2734, demonstrated strong antitumor activity by suppressing KRAS membrane localization and inducing apoptosis in KRAS-dependent tumors, representing a promising dual-targeting candidate (Kazi et al., 2019). Parallel approaches to disrupt the mevalonate pathway, which generates prenyl donors, include statins, zoledronic acid (ZA), and novel allosteric farnesyl pyrophosphate synthetase (FPPS) inhibitors, though limitations such as poor pharmacokinetics and bone affinity have restricted translation (Gnant et al., 2009; Jahnke et al., 2010; Senaratne et al., 2002). Beyond prenylation, targeting post-prenylation enzymes has also gained attention. RAS-converting enzyme 1 (RCE1) inhibitors like NSC1011 and its SAR-derived analogs can mislocalize KRAS, but genetic evidence raises concerns about cardiotoxicity and oncogenic exacerbation (Manandhar et al., 2010; Mohammed et al., 2016). In contrast, isoprenylcysteine carboxyl methyltransferase (ICMT) inhibitors have shown more substantial promise. Cysmethynil, an indole derivative, effectively disrupts KRAS localization, induces autophagy, and suppresses xenografts, although its poor solubility limits development (Wang et al., 2008; Winter-Vann et al., 2005). The optimized derivative compound 8.12 improves pharmacokinetic properties while retaining antitumor efficacy (Lau et al., 2014). Beyond these core membrane-targeting modifications, alternative strategies have emerged, such as employing the protein kinase C (PKC) agonist bryostatin-1 to stimulate KRAS phosphorylation on Serine-181, which promotes its dissociation from the plasma membrane and exerts antitumor effects (Bivona et al., 2006). Meanwhile, targeting the ubiquitin–proteasome system has emerged as another direction, with engineered ubiquitin ligases and chimeric constructs showing potential for selective KRAS degradation in pancreatic cancer models (Ma et al., 2013; Pan et al., 2016). Finally, other PTMs such as acetylation, palmitoylation, nitrosylation, and sumoylation remain under investigation as additional regulatory checkpoints (Ahearn et al., 2018; Campbell and Philips, 2021).

However, while these inhibitors have shown encouraging clinical efficacy, they are not without limitations. The clinical deployment of KRAS G12C inhibitors has delineated a class-specific toxicity profile characterized by on-target, off-tumor gastrointestinal (GI) effects and clinically significant organ-specific adverse events that necessitate vigilant, agent-specific monitoring. The most common adverse events are mechanism-based, arising from inhibition of wild-type RAS signaling, and are dominated by gastrointestinal toxicities. Adverse reactions have been commonly observed in clinical trials of sotorasib and adagrasib (Table 2) (Ou et al., 2022; Skoulidis et al., 2021). For sotorasib, pivotal studies reported diarrhea, nausea, vomiting, and hepatotoxicity as frequent adverse events (AEs), with transaminase elevations (aspartate Aminotransferase (AST)/alanine Aminotransferase (ALT)) representing the leading grade ≥3 events that often required dose reduction or treatment interruption (Hong et al., 2020; Skoulidis et al., 2021). Adagrasib exhibits a similar toxicity spectrum but is associated with a somewhat broader AE profile, including fatigue, decreased appetite, dehydration, and QT prolongation, in addition to gastrointestinal and hepatic events (Bekaii-Saab et al., 2023; Jänne et al., 2022). Importantly, interstitial lung disease (ILD)/pneumonitis, though infrequent, has been documented with both drugs and warrants close clinical monitoring (Hong et al., 2020; Jänne et al., 2022). Recently, a cross-comparison using Venn analysis further underscored the overlap and distinctions in their safety profiles, identifying 19 common AEs across four algorithms (Wu et al., 2024). Quantitatively, the analysis showed that sotorasib carried stronger signals for hepatotoxicity, liver enzyme abnormalities (increased AST, ALT, and gamma-glutamyl transferase (GGT)), and decreased appetite, whereas adagrasib demonstrated higher reporting odds for vomiting, systemic decline, death, neoplasm progression, and pneumonitis (Frey, 2025; Wu et al., 2024). In a focused cohort study, hepatotoxicity was observed in 65% of patients receiving sotorasib, with 31% developing severe cases, typically within 2 months of therapy initiation (Chour et al., 2023). Importantly, risk was highest among those recently treated with anti-programmed cell death ligand 1 (PD-L1) therapy, where severe hepatotoxicity occurred in 83% of patients starting sotorasib within 6 weeks of immunotherapy, compared to 13% when the interval exceeded 12 weeks (Chour et al., 2023). These findings underscore the need for proactive liver-function monitoring, careful sequencing with immunotherapy, and early management of gastrointestinal and respiratory toxicities during KRAS G12C inhibitor therapy. Early data on next-generation inhibitors like Divarasib (GDC-6036) suggest a consistent pattern of low-grade GI and hepatic events with 11% grade 3 adverse events and no new safety signals (Brazel and Nagasaka, 2024), while JDQ443 has shown acceptable early tolerability (Cassier et al., 2023). Taken together, these findings indicate that while first-generation KRAS G12C inhibitors are limited by gastrointestinal, hepatic, and immunotherapy-related toxicities, emerging next-generation agents may offer improved tolerability with fewer high-grade events and no unexpected safety concerns. Continued long-term follow-up and real-world data will be essential to confirm whether these agents can sustain efficacy while further reducing the toxicity burden associated with KRAS-targeted therapy.

**TABLE 2:** Overview of FAD-approved KRAS inhibitors.

Drug	Approval Date	Clinical Indications	Adverse Reactions (≥20%)	Laboratory Abnormalities (≥25%)	Recommended Dose
Sotorasib (AMG510)	5.28.2021	Adult patients with KRAS G12C-mutated locally advanced or metastatic non-small cell lung cancer (NSCLC)	Diarrhea, musculoskeletal pain, nausea, fatigue, hepatotoxicity, and cough	Decreased lymphocytes, hemoglobin, calcium, and sodium; elevated aspartate aminotransferase (AST), alanine aminotransferase (ALT), alkaline phosphatase, and urine protein	960 mg orally once daily, with or without food
Adagrasib (MRTX849)	12.12.2022	Adult patients with KRAS G12C-mutated locally advanced or NSCLC.	Diarrhea, nausea, fatigue, vomiting, musculoskeletal pain, hepatotoxicity, renal impairment, dyspnea, edema, decreased appetite, cough, pneumonia, dizziness, constipation, abdominal pain, and QTc interval prolongation	Decreased lymphocytes, sodium, hemoglobin, platelets, magnesium, and potassium; elevated albumin, creatinine, aspartate aminotransferase (AST), alanine aminotransferase (ALT), and lipase	600 mg orally twice daily until disease progression or unacceptable toxicity

### 2.3 Latest development of KRAS inhibitors for cancer therapy

As the field of KRAS inhibitors enters a period of rapid growth, several challenges have emerged, including clinical side effects and the development of resistance. Understanding the mechanisms underlying resistance is critical for improving therapeutic outcomes. Research has identified multiple factors contributing to acquired resistance, such as single-residue mutations, high KRAS G12C allele expression, activation of hepatocyte growth factor receptor (HGFR), NRAS isoform upregulation, and rapid reactivation of upstream signaling pathways (Nussinov and Jang, 2024; Singhal et al., 2024). Additionally, biomarkers associated with resistance to Sotorasib in KRAS G12C-mutated lung adenocarcinoma, including solute carrier family 2 member 1 (SLC2A1), transducin-like enhancer protein 1 (TLE1), family with sequence similarity 83 member A (FAM83A), high mobility group AT-hook 2 (HMGA2), F-box protein 44 (FBXO44), and MT-RNR2-like 12 (MTRNR2L12), are linked to abnormal PD‐L1 expression (Lin et al., 2024). These findings provide valuable insights into potential resistance mechanisms and offer new avenues for therapeutic intervention. Consistent with these insights, PTM-directed agents are also increasingly explored as rational partners in combination regimens to prevent or delay adaptive reactivation of KRAS signaling.

To address these challenges, researchers are exploring innovative strategies, including the development of pan-RAS/KRAS inhibitors that target a broad spectrum of RAS mutations, including wild-type amplifications (Coley et al., 2022). For example, NST-628, a pan-RAF-MEK non-degrading molecular glue, is a potent and brain-penetrant inhibitor of the RAS-MAPK pathway with activity across diverse RAS- and RAF-driven cancers (Ryan et al., 2024). Currently in phase 1 clinical trials (NCT06326411), NST-628 shows promise but may also pose toxicity risks to normal tissues (Ryan et al., 2024).

Combination therapies have also gained significant attention as a strategy to overcome resistance and enhance treatment efficacy. Ongoing and early-phase combination strategies can be categorized into four main categories: vertical inhibition, inhibition of protective adaptive responses, co-targeting distal RAS effectors, and capitalizing on other cancer-associated vulnerabilities (Perurena et al., 2024). For instance, combining Sotorasib with a potent SOS1 proteolysis-targeting chimeras (PROTAC) degrader has shown synergistic effects against KRAS G12C-mutant cells in preclinical models (Lv et al., 2024). In clinical studies, the combination of sotorasib with panitumumab, EGFR inhibitor, demonstrated acceptable safety and promising efficacy in chemotherapy-refractory KRAS G12C-mutated metastatic colorectal cancer (Kuboki et al., 2024). Other encouraging combinations include sotorasib with HRX0233 (a MAP2K4 inhibitor) and sotorasib with tipifarnib (a farnesyltransferase inhibitor) (Baranyi et al., 2024; Jansen et al., 2024; Kavgaci et al., 2024).

While combination therapies hold great potential for reducing resistance and improving outcomes, they also raise concerns about increased toxicity. Balancing efficacy and safety remains a critical challenge in the development of next-generation KRAS-targeted therapies. Ongoing research continues to drive advancements in KRAS inhibition, with multiple Phase III clinical trials evaluating both monotherapies and combination approaches in advanced cancers (Table 3). The development pipeline for next-generation RAS inhibitors is rapidly progressing beyond early-phase studies, with promising agents now entering late-stage clinical evaluation, particularly in advanced disease settings. The integration of combination strategies, including chemotherapy, immunotherapy, and targeted therapies, is further expanding the therapeutic landscape of RAS-driven cancers. These efforts underscore the ongoing commitment to optimizing RAS inhibition, refining treatment strategies, and addressing the clinical challenges associated with KRAS-targeted therapy.

**TABLE 3:** Representative recruiting and active phase III clinical trials for KRAS G12C inhibitors.

Inhibitor	Combination	Sponsor	Study title	Conditions	Objective	NCT number
Divarasib (GDC-6036)	Pembrolizumab	Roche	A Study Evaluating the Efficacy and Safety of Divarasib and Pembrolizumab Versus Pembrolizumab and Pemetrexed and Carboplatin or Cisplatin in Participants With Previously Untreated, KRAS G12C-Mutated, Advanced or Metastatic Non-Squamous Non-Small Cell Lung Cancer	KRAS G12C Lung Cancer, Non-Small Cell Lung Cancer (NSCLC)	Efficacy, Safety	NCT06793215
Divarasib (GDC-6036)		Roche	A Study Evaluating the Efficacy and Safety of Divarasib Versus Sotorasib or Adagrasib in Participants With Previously Treated KRAS G12C-positive Advanced or Metastatic Non-Small Cell Lung Cancer	KRAS G12C Lung Cancer, NSCLC	Compare Efficacy of Drug	NCT06497556
Divarasib (GDC-6036)		Roche	A Study to Evaluate the Efficacy and Safety of Multiple Targeted Therapies as Treatments for Participants with Non-Small Cell Lung Cancer (NSCLC) (B-FAST)	NSCLC	Compare Efficacy and Safety of Multiple Targeted Therapies	NCT03178552
RMC-6236		Revolution Medicines	Phase 3 Study of RMC-6236 in Patients with Previously Treated Metastatic Pancreatic Ductal Adenocarcinoma (PDAC)	PDAC, Pancreatic Cancer	Efficacy	NCT06625320
D-1553		Hansoh Pharmaceutical Group	D-1553 Tablet Versus Docetaxel Injection for KRAS G12C Mutation-positive Locally Advanced or Metastatic Non-small Cell Lung Cancer After Prior Standard Therapy Failure	NSCLC	Compare D-1553 with Docetaxel	NCT06300177
JDQ443		Novartis Pharmaceuticals	Study of JDQ443 in Comparison With Docetaxel in Participants With Locally Advanced or Metastatic KRAS G12C Mutant Non-small Cell Lung Cancer	NSCLC	Compare Efficacy	NCT05132075
Selumetinib	Docetaxel	AstraZeneca	Assess Efficacy and Safety of Selumetinib in Combination With Docetaxel in Patients Receiving 2nd Line Treatment for KRAS Positive NSCLC	Locally Advanced or Metastatic NSCLC Stage IIIb - IV	Efficacy, Safety	NCT01933932
MK-1084	Pembrolizumab	Merck Sharp and Dohme	A Study of MK-1084 Plus Pembrolizumab (MK-3475) in Participants With KRAS G12C Mutant, Metastatic Non-small Cell Lung Cancer (NSCLC) With Programmed Cell Death Ligand 1 (PD-L1) Tumor Proportion Score (TPS) ≥50% (MK-1084–004)	KRAS G12C-Mutant, Metastatic NSCLC With PD-L1 TPS ≥50%	Efficacy, Safety	NCT06345729
Sotorasib (AMG 510)		Amgen	Study of Sotorasib, Panitumumab and FOLFIRI Versus FOLFIRI With or Without Bevacizumab-awwb in Treatment-naïve Participants With Metastatic Colorectal Cancer With KRAS p.G12C Mutation (CodeBreaK 301)	Metastatic Colorectal Cancer	Compare Progression Free Survival Among Drugs	NCT06252649
Sotorasib (AMG 510)	Pembrolizumab	Amgen	A Study Evaluating Sotorasib Platinum Doublet Combination Versus Pembrolizumab Platinum Doublet Combination as a Front-Line Therapy in Participants With Stage IV or Advanced Stage IIIB/​C Nonsquamous Non-Small Cell Lung Cancers (CodeBreaK 202)	Stage IV or Advanced Stage IIIB/C Nonsquamous NSCLC	Efficacy	NCT05920356
Sotorasib (AMG 510)	Pembrolizumab	Amgen	Sotorasib and Panitumumab Versus Investigator’s Choice for Participants With Kirsten Rat Sarcoma (KRAS) p.G12C Mutation (CodeBreak300)	Metastatic Colorectal Cancer	Compare Progression Free Survival Among Drugs	NCT05198934
Sotorasib (AMG 510)	Docetaxel	Amgen	A Phase 3 Study to Compare AMG 510 with Docetaxel in Non Small Cell Lung Cancer (NSCLC) subjects with KRAS p. G12c mutation	Advanced and Unresectable or Metastatic NSCLC	Efficacy	NCT04303780
Olomorasib (LY3537982)	Pembrolizumab	Eli Lilly	A Study of First-Line Olomorasib (LY3537982) and Pembrolizumab With or Without Chemotherapy in Patients With Advanced KRAS G12C-Mutant Non-small Cell Lung Cancer (SUNRAY-01)	Carcinoma, NSCLC, Neoplasm Metastasis	Efficacy, Safety	NCT06119581
Adagrasib (MRTX849)	Cetuximab, mFOLFOX6 Regimen, FOLFIRI Regimen	Mirati Therapeutics	Phase 3 Study of MRTX849 With Cetuximab vs. Chemotherapy in Patients With Advanced Colorectal Cancer With KRAS G12C Mutation (KRYSTAL-10)	Advanced Colorectal Cancer, Metastatic Colorectal Cancer	Efficacy	NCT04793958
Adagrasib (MRTX849)		Mirati Therapeutics	Phase 3 Study of MRTX849 (Adagrasib) vs. Docetaxel in Patients With Advanced Non-Small Cell Lung Cancer With KRAS G12C Mutation (KRYSTAL-12)	Metastatic and Advanced NSCLC	Efficacy	NCT04685135
Adagrasib (MRTX849)	Pembrolizumab	Mirati Therapeutics	Phase 2 Trial of Adagrasib Monotherapy and in Combination With Pembrolizumab and a Phase 3 Trial of Adagrasib in Combination in Patients With a KRAS G12C Mutation KRYSTAL-7	Advanced NSCLC	Compare the efficacy of Treatment	NCT04613596

### 2.4 Clinical translation and therapeutic context of RAS inhibition

The transition of RAS inhibitors from preclinical promise to clinical reality represents a watershed moment in oncology, yet it has unveiled a new set of complexities that define their modern therapeutic application. While the approval of allele-specific inhibitors validates RAS as a druggable target, their clinical utility is not universal but is instead governed by a sophisticated interplay of molecular biomarkers, tissue-specific vulnerabilities, and a dynamic tumor microenvironment.

#### 2.4.1 Pan-cancer applicability

KRAS is one of the most frequently mutated oncogenes across solid tumors. It is nearly universal in pancreatic ductal adenocarcinoma, highly prevalent in colorectal and NSCLC, and present in biliary tract, ovarian, and endometrial malignancies (Moore et al., 2020; Prior et al., 2020). Allele distributions vary by tissue type, with G12D/G12V most common in pancreatic and colorectal cancers, while G12C is more frequently in lung adenocarcinoma (Cox et al., 2014; Hobbs et al., 2016). This variability supports both tumor-specific and tumor-agnostic development and provides the rationale for pursuing allele-specific as well as pan-KRAS therapeutic strategies.

#### 2.4.2 Cancer-specific considerations

The efficacy of KRAS-targeted therapies varies considerably by tumor type, reflecting differences in co-mutation landscapes, signaling dependencies, and tumor biology. In NSCLC, both sotorasib and adagrasib have demonstrated the most robust single-agent activity, with objective response rates (ORR) of approximately 37%–43% (Jänne et al., 2022; Skoulidis et al., 2021). In contrast, colorectal cancer (CRC) shows limited benefit to monotherapy (ORR <10%), largely due to rapid EGFR-driven pathway reactivation. This limitation has been overcome through combination strategies where the phase 3 CodeBreaK-300 trial showed that sotorasib plus panitumumab outperformed standard therapy (Fakih et al., 2023), and adagrasib with cetuximab subsequently secured FDA approval based on KRYSTAL-1 (Yaeger et al., 2024). Compared with NSCLC, in pancreatic and biliary tract cancers, efficacy has been more modest, with early trials reporting ORRs of approximately 20% and 10%, respectively (Bekaii-Saab et al., 2023; Hong et al., 2020). Preclinical studies suggest that stromal barriers and compensatory signaling may contribute to this reduced sensitivity (Moore et al., 2020; Ryan and Corcoran, 2018). Additional clinical considerations include central nervous system involvement, where adagrasib has demonstrated intracranial penetration and activity in NSCLC patients with brain metastases (Jänne et al., 2022).

#### 2.4.3 Key biomarkers for patient selection

Recent evidence suggests that allele-specific expression levels and genomic context may further refine patient selection. Patient selection for KRAS-targeted therapy relies foremost on the presence of a defined mutation, with KRAS G12C serving as the key biomarker for current inhibitors such as sotorasib and adagrasib (Hong et al., 2020; Jänne et al., 2022). Efforts to expand the therapeutic reach have led to the development of pan-KRAS inhibitors that can target multiple alleles, including G12D, G12V, G13D, and G12R, as well as amplified wild-type KRAS (Moore et al., 2020). Predictive markers further refine expectations of response, as in NSCLC where high thyroid transcription factor 1 (TTF-1) expression is associated with improved survival, while kelch-like ECH-associated protein 1 (KEAP1) and serine/threonine kinase 11 (STK11) mutations consistently signal resistance and poor prognosis (Arbour et al., 2018); TP53 alterations appear more prognostic than predictive but may shape outcomes with immunotherapy (Arbour et al., 2018). Functional and dynamic markers are also emerging, including the degree of RAS-RAF interaction (Kato et al., 2025) and clearance of circulating tumor DNA (ctDNA) (Ernst et al., 2024), of which correlate with treatment benefit. In CRC, KRAS mutations confer resistance to EGFR inhibitors, but KRAS G12C blockade combined with EGFR targeting has proven effective (Fakih et al., 2023; Yaeger et al., 2024).

#### 2.4.4 Contextual dependencies/tumor microenvironment

The efficacy of KRAS inhibitors is heavily contingent on contextual dependencies within the tumor microenvironment, which not only modulate initial responses but also actively drive resistance. Oncogenic KRAS frequently engenders an immunosuppressive milieu through recruitment of suppressive immune subsets such as myeloid-derived suppressor cells (MDSCs), tumor-associated macrophages (TAMs), and regulatory T cells. It also downregulates antigen presentation pathways. Co-mutations such as STK11/LKB1 and KEAP1 correlate with “immune-cold” phenotypes and poor responses to immune checkpoint inhibitors in lung adenocarcinoma. In KRAS-mutant non-small cell lung cancer, STK11 co-mutations are linked to reduced CD8^+^ T-cell infiltration and diminished PD-L1 expression. By contrast, TP53 co-mutations promote higher tumor mutational burden and a more inflamed microenvironment (Ricciuti et al., 2022; Schabath et al., 2016). In PDAC, KRAS inhibitors can partially remodel the microenvironment by reducing myeloid cell accumulation, increasing CD8^+^ T-cell infiltration, and reprogramming cancer-associated fibroblasts (Mahadevan et al., 2023). However, adaptive resistance frequently develops through stromal feedback and immune evasion mechanisms (Hosein et al., 2020; Tape et al., 2016). These dependencies highlight the importance of combining KRAS inhibitors with immunotherapy, stromal-modulating agents, or co-mutation–directed strategies to overcome context-specific resistance. These dependencies highlight the need for rational combinations of KRAS inhibitors with immunotherapy, stromal-modulating agents, or agents that target co-mutations to overcome context-specific resistance.

The clinical translation of RAS inhibitors marks a pivotal step in precision oncology, showing that success depends on factors beyond a single oncogenic driver. Therapeutic efficacy is shaped by co-mutations, tissue-specific adaptations, and the tumor microenvironment, emphasizing that RAS-driven cancers represent a spectrum of distinct vulnerabilities. The future of RAS inhibition lies in biomarker-guided strategies, rational drug combinations, and deeper insight into tumor-microenvironment interactions. The journey to effectively neutralize this once “undruggable” target continues to reveal that the greatest challenges and opportunities lie in the nuances of clinical application.

## 3 Targeting the PNCA pathway: a potential pan-cancer target

### 3.1 PCNA: biology, function, and clinical relevance in cancer

PCNA is a highly conserved protein that regulates DNA replication and the cell cycle in eukaryotic and archaeal cells (Strzalka and Ziemienowicz, 2011). PCNA was first identified in 1978 when the serum of systemic lupus erythematosus patients exhibited an autoantibody reacting with proliferating cells’ nuclear antigens (Miyachi et al., 1978). After significant analysis, the structure and function of PCNA were determined to be stable despite millions of evolutionary years (Miyachi et al., 1978; Strzalka and Ziemienowicz, 2011). The structural characterization of PCNA progressed with the publication of the yeast PCNA crystal structure in 1994 (Krishna et al., 1994), followed 2 years later by the crystal structure of human PCNA complexed with a fragment of the cell-cycle regulator p21(WAF1/CIP1), catalyzing future research on the protein’s function and clinical potential (Gulbis et al., 1996).

PCNA is categorized as a DNA sliding clamp, and its 87 kDa homotrimeric ring encircles DNA, providing a stable platform for the recruitment and retention of replication polymerases δ and ε at the replication fork (González-Magaña and Blanco, 2020). This ability to secure polymerases ensures continuous DNA synthesis and high replication fidelity. PCNA is also essential for lagging strand synthesis, where it facilitates Okazaki fragment maturation by coordinating the activity of polymerase δ, flap endonuclease 1 (FEN1), and DNA ligase 1 (LIG1), ensuring the proper processing and ligation of DNA fragments (González-Magaña and Blanco, 2020; Krishna et al., 1994). The evolutionary conservation of PCNA across eukaryotes and archaea underscores its indispensable role in maintaining genome stability (Moldovan et al., 2007). Additionally, PCNA is integral to DNA repair and cell cycle control (Strzalka and Ziemienowicz, 2011). Homologous recombination requires PCNA to promote polymerase and nuclease processivity for DNA repair synthesis and resection (Slade, 2018). PCNA also plays a crucial role in base excision repair, nucleotide excision repair, and mismatch repair, where it coordinates the recruitment of specific repair enzymes to damaged sites (Slade, 2018). Alongside DNA replication and repair, PCNA contributes to the regulation of chromatin structure and gene expression (Wang et al., 2022). By recruiting chromatin assembly factors, it facilitates histone deposition and nucleosome reassembly after DNA replication and repair, ensuring the preservation of epigenetic memory across cell generations (Wang et al., 2022).

PCNA’s diverse cellular functions are tightly regulated through post-translational modifications that modulate its interactions with various protein partners (González-Magaña and Blanco, 2020). One well-studied modification is phosphorylation at tyrosine 211 (Y211), which has been linked to cancer progression by promoting tumor cell proliferation and invasion (Wang et al., 2022). Aberrant phosphorylation of PCNA at Y211 has been identified in several malignancies and is associated with poor patient prognosis (Zhang et al., 2021). Additionally, other phosphorylation sites have been discovered and are actively studied for their roles in regulating PCNA activity in cancer pathogenesis (González-Magaña and Blanco, 2020; Moldovan et al., 2007). Apart from phosphorylation, PCNA undergoes ubiquitination and SUMOylation, modifications that influence its role in DNA repair and damage tolerance mechanisms (Maga and Hubscher, 2003). Further, PCNA binds numerous proteins, which enhances its functional capabilities. Many of its interacting partners belong to the class of intrinsically disordered proteins (IDPs), which lack stable secondary and tertiary structures but play crucial roles in cell cycle progression, apoptosis, and genomic maintenance (González-Magaña and Blanco, 2020). These proteins bind to PCNA with a specific PCNA interacting protein-box (PIP box) that was first characterized using the crystal structure of human PCNA bound to a p21 fragment (González-Magaña and Blanco, 2020). One notable example is PCNA‐associated factor p15, an IDP that is overexpressed in cancer cell nuclei and mitochondria, where its elevated levels correlate with poor prognosis in several human cancers (González-Magaña and Blanco, 2020).

**FIGURE 3:**
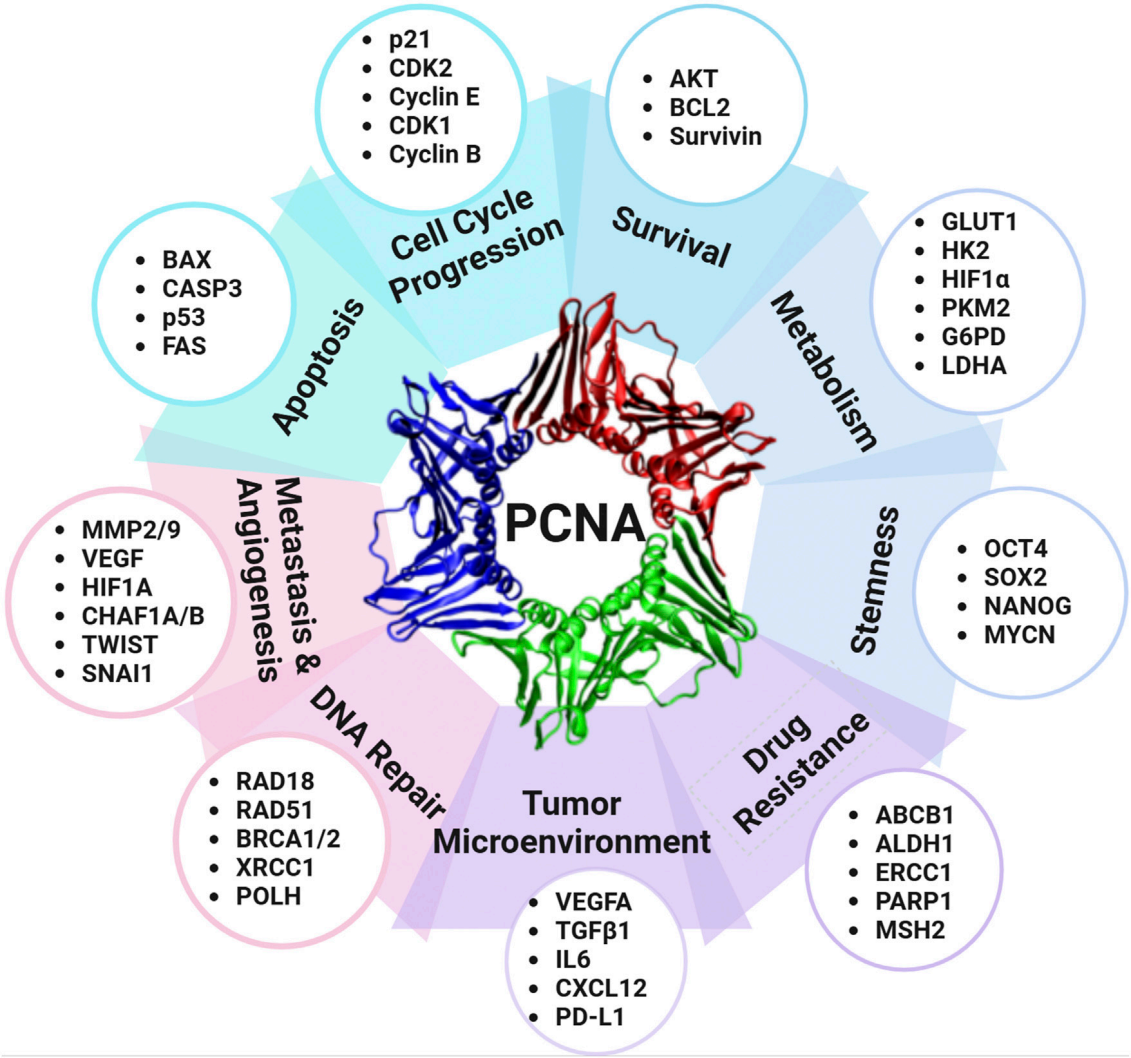
PCNA as a central regulator of cancer pathways. PCNA regulates a variety of cellular proteins and contributes to multiple processes of carcinogenesis and cancer progression, including cell cycle regulation, survival, metabolism, stemness, drug resistance, tumor microenvironment modulation, DNA repair, metastasis, angiogenesis, and apoptosis. Abbreviations: ABCB1: ATP-binding cassette subfamily B member 1; ALDH1: Aldehyde dehydrogenase 1; AKT: Protein kinase B; BAX: BCL2-associated X protein; BCL2: B-cell lymphoma 2; BRCA1/2: Breast cancer susceptibility genes 1 and 2; CASP3: Caspase-3; CDK1: Cyclin-dependent kinase 1; CDK2: Cyclin-dependent kinase 2; CHAF1A/B: Chromatin assembly factor 1 subunit A/B; CXCL12: C-X-C motif chemokine ligand 12; ERCC1: Excision repair cross-complementation group 1; FAS: Fas cell surface death receptor; G6PD: Glucose-6-phosphate dehydrogenase; GLUT1: Glucose transporter 1; HIF1A: Hypoxia-inducible factor 1-alpha; HK2: Hexokinase 2; IL6: Interleukin 6; LDHA: Lactate dehydrogenase A; MMP2/9: Matrix metalloproteinases 2 and 9; MSH2: MutS homolog 2; MYCN: MYCN proto-oncogene; OCT4: Octamer-binding transcription factor 4; PARP1: Poly (ADP-ribose) polymerase 1; PD-L1: Programmed death-ligand 1; PKM2: Pyruvate kinase M2; POLH: DNA polymerase eta; NAI1: Snail family transcriptional repressor 1; SOX2: SRY-box transcription factor 2; SURVIVIN (BIRC5): Baculoviral IAP repeat-containing protein 5; TGFβ1: Transforming growth factor-beta 1; TWIST: Twist family bHLH transcription factor; VEGFA: Vascular endothelial growth factor A; XRCC1: X-ray repair cross-complementing protein 1.

PCNA plays a crucial role in DNA replication, repair, and cell cycle regulation, which has led to its recognition as an important biomarker in oncology, particularly regarding tumor development and cancer progression (Figure 3). With the ability of cancer cells to evade cell cycle regulation and apoptosis, PCNA overexpression is commonly observed in various malignancies, including breast cancers (Malkas et al., 2006; Smith et al., 2015), duodenal cancers (Imazu et al., 1992), non-small cell lung cancer (NSCLC) (Lamort et al., 2022; Ye et al., 2020), liver cancer (Li et al., 2020; Zheng et al., 2019), nasal and paranasal sinus cancers (Mumbuc et al., 2007), and colorectal cancer (Kasprzak, 2023). This upregulation often translates to poorer clinical outcomes, as higher PCNA levels have been linked to increased tumor proliferation, enhanced metastatic potential, and reduced patient survival. Specifically in NSCLC, PCNA protein level was found to be significantly higher in the cancerous tissues than in the adjacent tissues, with increased PCNA levels correlating with shorter disease-specific survival (Ye et al., 2020). Another study considering the resected, early-stage lung adenocarcinoma analyzed PCNA as part of a prognostic phenotype and concluded that higher PCNA expression had statistically significant decreased 5-year overall survival (Lamort et al., 2022). In hepatocellular carcinoma, PCNA, along with cell cycle regulators GTSE1, CDC20, and MCM6, was found to drive tumor progression and predict poor prognosis, establishing PCNA as a potential molecular biomarker for liver cancer (Zheng et al., 2019). Additionally, PCNA serves as an immunohistochemical proliferative marker with prognostic significance in colorectal cancer, providing insight into both overall and disease-free survival rates (Kasprzak, 2023). Beyond protein expression, genomic profiling reveals that PCNA is subject to diverse alterations across human cancers (Table 4). The strong association between PCNA expression and cancer progression has driven extensive research into its therapeutic potential, with PCNA-targeted therapies under investigation to enhance treatment outcomes across various cancers.

**TABLE 4:** PCNA alteration frequencies across human cancers.

Cancer types	Total rate (%)	Alteration (%)		
		Mutation	Amplication	Deep deletion
Lung Cancer	10.52	0.00	7.89	2.63
Esophagogastric Cancer	9.88	0.00	9.58	0.3
Uterine Endometrioid Carcinoma	8.33	0.00	8.33	0.00
Ovarian Cancer	5.13	0.00	5.13	0.00
Renal Cell Carcinoma	3.14	0.7	2.44	0.00
Bone Cancer	2.88	0.00	1.92	0.96
Cervical Cancer	2.70	0.00	2.7	0.00
Melanoma	2.65	0.57	2.08	0.00
Head and Neck Cancer	2.60	0.00	2.6	0.00
Pancreatic Cancer	2.50	0.00	2.41	0.09
Hepatobiliary Cancer	2.17	0.00	2.17	0.00
Breast Cancer	2.01	0.00	2.01	0.00
Colorectal Cancer	1.97	0.22	1.75	0.00
Bladder Cancer	1.56	0.39	1.17	0.00
Embryonal Tumor	0.74	0.00	0.74	0.00
Non-Small Cell Lung Cancer	0.54	0.09	0.45	0.00
Endometrial Cancer	0.39	0.00	0.39	0.00
Soft Tissue Sarcoma	0.36	0.00	0.24	0.12
Leukemia	0.34	0.00	0.00	0.34
Glioma	0.29	0.29	0.00	0.00
Wilms Tumor	0.15	0.00	0.15	0.00
Prostate Cancer	0.15	0.00	0.00	0.15

Data sources from cBioPortal.org (date to 11 Sep 2025). Total cases were more than 10 are listed.

### 3.2 Targeting PCNA: challenges, failures, and breakthroughs

PCNA is a well-established target in cancer therapy due to its critical involvement in cell proliferation, DNA replication, and repair. However, drug discovery efforts focused on PCNA have encountered significant challenges, leading many researchers to label it as “undruggable” (Choe and Moldovan, 2017; Wang and Wang, 2025). The primary challenge lies in the absence of known endogenous small-molecule modulators and well-defined ligand-binding sites (Wang and Wang, 2025). From a structural and medicinal chemistry perspective, PCNA lacks conventional binding grooves and instead presents relatively small and shallow surface pockets, which hinder the discovery of high-affinity inhibitors (Wang and Wang, 2025). Its interactions with replication and repair proteins rely on transient and flexible binding motifs, making it difficult to develop stable inhibitors that effectively interfere with its function (Wang and Wang, 2025). In addition, medicinal chemistry hurdles remain formidable. Many small-molecule inhibitors, such as T2AA, have suffered from weak affinity and poor solubility, while more advanced derivatives like AOH1160 improved potency but retained solubility and toxicity concerns (Gu et al., 2023a; Punchihewa et al., 2012). In addition, the promiscuity of the PIP-box binding groove further complicates inhibitor design. With hundreds of human proteins containing this motif, any agent that non-selectively blocks this interface would disrupt essential replication and repair machinery, resulting in catastrophic cellular toxicity (Choe and Moldovan, 2017; Prestel et al., 2019). Thus, the ultimate goal is not mere inhibition, but rather the exceptionally refined task of achieving selective disruption of cancer-specific PCNA interactions.

Biologically, PCNA is highly conserved between normal and malignant cells, creating a therapeutic paradox (Prestel et al., 2019). Inhibitors must block tumor proliferation while sparing vital functions in normal proliferative tissues, including the bone marrow and gastrointestinal tract. This lack of selectivity predicts severe on-target toxicities, resembling the adverse effects of conventional chemotherapies. Moreover, PCNA’s indispensable role in DNA replication, DNA damage responses, and cell cycle regulation complicates therapeutic intervention, as systemic inhibition risks catastrophic impairment of normal cellular functions (Gu et al., 2023a; Prestel et al., 2019). Given its essential role in all proliferating cells, drug development has been further complicated by the need to achieve selective toxicity in cancer while sparing normal tissues.

#### 3.2.1 PCNA-targeting peptides

PCNA-targeting research initially focused on peptide-based drugs, but challenges in stability and systemic toxicity hindered their clinical application (Table 5). One of the first peptide inhibitors was a 39-amino acid fragment of p21, called p21C2, which bound to PCNA and inhibited DNA replication *in vitro*. However, its structural instability limited its therapeutic potential (Chen et al., 1996). Another early peptide, p21PBP, targeted the p21-PCNA interaction and was detailed by Warbrick and colleagues in 1995 (Warbrick et al., 1995). Further research explored post-translational modifications of PCNA to improve specificity. In 2012, researchers identified Y211 phosphorylation of PCNA as a marker for prostate cancer and developed a synthetic Y211F peptide to inhibit this modification, which enhanced cancer cell death (Zhao et al., 2011). While this strategy increased targeting specificity, the potential for systemic on-target toxicity remained a significant concern due to PCNA’s widespread expression and the difficulty of achieving tumor-specific delivery for peptides (Zhao et al., 2011). Another breakthrough in peptide research came with the development of R9-caPeptide, introduced in 2014 (Gu et al., 2014). This peptide mimics a critical sequence in the cancer-associated PCNA isoform (caPCNA), blocking PCNA chromatin and DNA polymerase δ binding, leading to cytotoxicity in triple-negative breast cancer cells (Smith et al., 2015). The most recent development in peptide PCNA-inhibitors highlighted Con1-SPOP, a biodegrader of PCNA, that had anti-proliferative effects and significantly decreased PCNA in hours when delivered via lipid nanoparticle (Chang et al., 2022). Unfortunately, *in vivo* delivery of biodegraders is not yet sufficient for clinical trials (Chang et al., 2022).

**TABLE 5:** Representative PCNA-targeting peptides and small molecule inhibitors.

Peptide/SMIs	Mechanism of action	Cancer type/Model	Limitations	References
PCNA-Targeting Peptides				
p21-Derived Peptide (p21C2)	Binds PCNA and inhibits DNA replication	*In vitro* models	Poor stability, undefined structure	Chen et al. (1996)
p21PBP	Targets p21-PCNA interaction to disrupt replication	*In vitro* models	Low specificity, stability issues	Warbrick et al. (1995)
Y211F-Peptide	Blocks Y211 phosphorylation of PCNA	Prostate cancer	Potential off-target effects	Zhao et al. (2011)
R9-caPep	Blocks PCNA interactions, interferes with DNA synthesis, impairs DNA repair	Neuroblastoma; Breast and Pancreatic cancer	Limited *in vivo* validation	Gu et al. (2014), Smith et al. (2015), Smith et al. (2020)
NKp44-Peptide (pep8)	interacts with PCNA and partly blocks the NKp44–PCNA interaction	Various solid tumors	Limited preclinical validation	Shemesh et al. (2018)
PIP-Box Peptides	Mimics PIP-box interactions, disrupts PCNA-protein binding	Multiple cancer types	Low stability and cellular permeability	Horsfall et al. (2021)
Con1-SPOP	Biodegrades PCNA, inhibits proliferation	Multiple cancer models	Challenges in *in vivo* delivery	Chang et al. (2022)
APIM-Peptide (ATX-101)	Targets APIM motif, impairs PCNA-dependent repair and metabolism	Various solid tumors, clinical trials (Phase II)	Potential systemic toxicity	Gravina et al. (2022), Lemech et al. (2023), Müller et al. (2013); ClinicalTrials.gov, (NCT05116683)
PCNA-Targeting Small Molecule Inhibitors				
PCNA-I	Stabilizes PCNA trimer, prevents loading onto DNA, disrupts replication	Various human and mouse cancer cells lines	Limited preclinical data available	Dillehay et al. (2015), Tan et al. (2012)
T2AA	inhibits PCNA/PIP-box peptide interaction, disrupts PCNA-Polδ3 and PCNA-p21 interaction	Preclinical studies	Possible off-target effects in normal cells	Punchihewa et al. (2012)
AOH1160	Targets the L126-Y133 region of caPCNA	Small cell lung cancer; preclinical animal models	Undesirable metabolic properties	Gu et al. (2018)
AOH1996	Improved caPCNA inhibitor targeting altered PCNA isoform	Refractory malignant solid neoplasms; Phase 1 clinical trials	Clinical trial ongoing; efficacy and safety under evaluation	Gu et al. (2023b); ClinicalTrials.gov, NCT05227326

One of the most advanced peptide-based PCNA inhibitors is ATX-101, a peptide derived from the AlkB homologue 2 PCNA-interacting motif (APIM) (Gravina et al., 2022). ATX-101 disrupts PCNA’s role in DNA repair and cell survival, selectively induces apoptosis in multiple myeloma cell lines and other cancer types, and sensitizes cancer cells to chemotherapy (Gravina et al., 2022; Müller et al., 2013). This strong rationale successfully translated into initial clinical encouragement during a Phase I study. Here, ATX-101 showed signals of disease control in a heterogeneous cohort of heavily pre-treated patients with advanced solid tumors, where 70% achieved stable disease (Lemech et al., 2023). The therapy was reported to be generally well-tolerated, with a safety profile primarily characterized by manageable, non-dose-limiting infusion-related reactions (IRRs) in 64% of patients. Critically, no traditional hematological or gastrointestinal dose-limiting toxicities (DLTs) were identified, and the maximum tolerated dose (MTD) was not reached (Lemech et al., 2023). However, the subsequent clinical development of ATX-101 starkly underscores the formidable challenge of translating an initially tolerable safety profile into a viable therapeutic window for a defined, refractory patient population. This favorable picture was dramatically contradicted by the outcomes of a focused Phase II trial (NCT05116683) in patients with locally advanced dedifferentiated liposarcoma and leiomyosarcoma that were metastatic or unresectable. Unfortunately, the study was terminated. Of the four patients enrolled, none completed the trial. The primary endpoint, assessing the 12-week progression-free survival rate using Response Evaluation Criteria in Solid Tumors (RECIST) criteria, could only be evaluated in three patients due to the death of one participant prior to week 12. Among these, only one patient achieved the primary outcome. Secondary analyses revealed that serious adverse events occurred in 1/4 patients (25%), including atrial fibrillation, acute kidney injury, dyspnea, and limb edema. Non-serious events were universal (4/4, 100%), dominated by infusion-related reactions despite prophylaxis (3/4, 75%) and anorexia (2/4, 50%); a wide spectrum of single-patient events (each 1/4, 25%) was also reported, including fatigue, generalized edema, dry mouth, nausea, vomiting, upper respiratory infection, hyperbilirubinemia, anemia, tumor pain, dysgeusia, urinary tract obstruction, cough, pleural effusion, periorbital edema, pruritus, and maculopapular rash. Most critically, the compound failed to demonstrate any meaningful efficacy, as no objective tumor responses were observed, only one of three evaluable patients met the primary endpoint of 12-week progression-free survival, and all four enrolled patients succumbed to rapid disease progression within the seven-month assessment period. While comprehensive clinical trial data remain undetailed and unpublished, this clinical outcome underscores the significant translational barriers facing PCNA inhibitor development. The study termination appears to have resulted from multiple contributing factors, including insufficient therapeutic efficacy that was possibly limited by the pharmacokinetic and solubility challenges inherent to peptide therapeutics, coupled with emerging mechanism-based toxicities. The serious adverse events observed, though documented in a small patient cohort, align with both predicted on-target effects on proliferating tissues and potential off-target toxicities. These findings highlight the narrow therapeutic index characterizing this drug class and emphasize the fundamental difficulty in achieving sufficient cancer cell selectivity while maintaining viability of essential normal proliferating tissues.

#### 3.2.2 PCNA-targeting small molecule inhibitors (SMI)

Targeting PCNA in cancer therapy has been a long-standing challenge, but recent advancements have opened new avenues for exploration. Other scientists approached the inhibition of PCNA by developing small molecule drugs (Table 5). In April of 2012, a non-peptide, small molecule PCNA inhibitor called T2 amino alcohol (T2AA) targeted PCNA protein interactions (Punchihewa et al., 2012). This molecule is a T3 derivative lacking thyroid hormone activity that interrupts PIP-box, p21, and DNA polymerase δ interactions with PCNA (Punchihewa et al., 2012). T2AA was found to arrest cells in the S-phase, induce early apoptosis, and increase replication stress (Punchihewa et al., 2012). However, this study was limited by T2AA lacking high PCNA affinity and complete cytotoxicity (Punchihewa et al., 2012). Following this attempt, scientists continued to pursue the development of PCNA SMIs.

Later, a class of PCNA inhibitors termed PCNA-Is emerged as potential anti-tumor agents (Tan et al., 2012). These compounds hindered PCNA’s role in DNA replication by selectively binding and stabilizing PCNA trimers, preventing chromatin-PCNA association. This mechanism induced cell cycle arrest in the S and G2/M phases, effectively reducing tumor proliferation (Tan et al., 2012). Among these, PCNA-I1 showed selective binding to PCNA trimers, reduced chromatin-associated PCNA, and suppressed tumor growth in various tissue types (Tan et al., 2012). These first-in-class compounds marked a significant step forward in PCNA-targeted cancer therapy.

A major breakthrough discovery identified a cancer-associated isoform of PCNA (caPCNA), which is highly expressed in tumor tissues but minimally present in normal cells (Malkas et al., 2006). This discovery revolutionized PCNA-targeted therapy by allowing selective inhibition of cancer cells, reducing off-target toxicity, and potentially minimizing acquired drug resistance (Malkas et al., 2006). Building on this discovery, AOH1160 was developed as a small molecule inhibitor targeting the L126-Y133 region of PCNA, a site altered in cancer cells that forms a unique binding pocket for proteins (Gu et al., 2018). By targeting this region, AOH1160 blocked PCNA interactions, leading to apoptosis in cancer cells due to failed DNA replication and repair. The drug demonstrated strong anti-tumor activity in preclinical models and was orally bioavailable in animals (Gu et al., 2018). Preclinical studies indicated that AOH1160 possessed selective anti-cancer activity with minimal toxicity to normal cells and showed no significant adverse effects at 2.5 times the effective dose in animal models (Gu et al., 2018). However, further investigation revealed suboptimal metabolic stability and pharmacokinetic properties of AOH1160, which precluded its advancement in the drug development pipeline (Gu et al., 2023b). To address these pharmacological limitations, researchers developed AOH1996 as a second-generation caPCNA inhibitor with enhanced drug-like properties (Gu et al., 2023b). Currently in Phase 1 clinical trials (NCT05227326), AOH1996 is being evaluated for its efficacy in treating refractory malignant solid neoplasms. The primary objectives are to determine the MTD, the incidence of AE, and DLT. The outcome of this trial is highly anticipated, as it will critically test whether the caPCNA targeting strategy can indeed mitigate the severe on-target toxicities that have plagued previous candidates. The DLT toxicities observed will be particularly informative, likely centering on hematological (neutropenia, thrombocytopenia) and gastrointestinal (diarrhea, mucositis) adverse events, which will define the practical therapeutic window for this modality. Secondary objectives include evaluating pharmacokinetics, efficacy, and solid tumor disease control and response rates. As this study progresses, the scientific community eagerly anticipates the results of these studies, which could validate the therapeutic potential of AOH1996 and transform PCNA from an “undruggable” target into a cornerstone of cancer therapy.

### 3.3 Clinical translation of PCNA-targeted therapies

The journey of PCNA from a compelling biological target to a viable therapeutic agent epitomizes the challenges of targeting fundamental biological processes in oncology. While its ubiquitous role in proliferation offers broad theoretical potential, translating this promise into safe and effective clinical strategies requires a deeper understanding of its toxicity profile, the identification of predictive biomarkers, and strategies to delineate tumor-specific dependencies.

#### 3.3.1 Pan-cancer applicability

The theoretical pan-cancer applicability of PCNA inhibition is exceptionally high, driven by the protein’s universal overexpression in malignancies and its fundamental role in DNA replication and repair processes essential for all proliferating cancer cells. PCNA is overexpressed across a wide spectrum of cancers, including breast, lung, liver, and colorectal malignancies, where its levels often correlate with poor prognosis (Pandit et al., 2025). This broad expression pattern suggests that effective PCNA inhibitors could have utility in numerous oncological indications. However, this very universality presents a significant challenge, as PCNA is equally critical for the function of normal, healthy proliferating tissues, inherently limiting the therapeutic window.

#### 3.3.2 Cancer-specific considerations

Not all cancers may be equally susceptible to PCNA-targeted therapy. The most promising applications are likely in cancers characterized by high replication stress, high proliferative indices, or those with specific dependencies on PCNA-mediated DNA repair pathways, such as triple-negative breast cancer, which tend to show stronger reliance on PCNA and thus greater vulnerability (Smith et al., 2015). Conversely, tumors with low proliferation rates or proficient DNA repair mechanisms may show resistance. The expression of specific cancer-associated PCNA (caPCNA) isoforms, which are not uniformly present across all malignancies, creates an additional layer of differential susceptibility, as inhibitors like AOH1996 are designed to target these variant forms (Gu et al., 2023b).

#### 3.3.3 Key biomarkers for patient selection

A validated biomarker for patient selection remains a critical unmet need in PCNA therapeutic development. The most straightforward biomarker is PCNA overexpression itself. The presence of the caPCNA isoform represents a more specific potential biomarker currently under investigation (Gu et al., 2023b). Beyond expression, functional dependencies in DNA repair are emerging. Phosphorylation of PCNA at tyrosine 211 (pY211) has been identified as a marker of cancer progression and could serve as a predictive biomarker for inhibitors targeting this modification (Wang YL. et al., 2021). Furthermore, in HCC, PCNA inhibition enhances sensitivity to the PARP inhibitor olaparib, with AOH1160 and olaparib showing synergistic anti-tumor effects *in vitro* and *in vivo*; elevated PCNA expression further correlated with poor prognosis, underscoring its potential to guide patient selection as both a therapeutic target and predictive biomarker (Li et al., 2025).

#### 3.3.4 Contextual dependencies/tumor microenvironment

The tumor microenvironment strongly influences the efficacy of PCNA inhibitors by regulating drug delivery, immune evasion, and resistance mechanisms. PCNA is aberrantly expressed on the tumor cell surface, where it binds the NKp44 receptor and suppresses natural killer (NK) cell activation, enabling immune escape (Rosental et al., 2011). Within tumor-associated macrophages (TAMs), high PCNA expression has been correlated with poor prognosis, suggesting a role in skewing macrophage function toward a pro-tumorigenic phenotype (Feng et al., 2019). Cytosolic PCNA also promotes neutrophil survival, fostering chronic inflammation that can sustain tumor growth (Bouayad et al., 2012). In the stromal compartment, phosphorylation of PCNA at Y211 in fibroblasts drives secretion of cytokines such as transforming growth factor beta (TGFβ) and C-X-C motif chemokine ligand 12 (CXCL12), which enhance cancer stemness and invasion potential (Wang et al., 2022). These findings demonstrate that PCNA is not only a replication factor but also a key modulator of TME dynamics, underscoring the need to integrate microenvironmental context into the design of PCNA-directed therapies.

The clinical development of PCNA inhibitors faces both promise and risk. PCNA remains an attractive target because of its central role in cancer proliferation, yet this same universality raises concerns about on-target toxicity in normal tissues. Future progress will depend on carefully defining clinical contexts where inhibition is most likely to succeed. Predictive biomarkers, such as the cancer-associated PCNA isoform and replication stress signatures, may help refine patient selection. Rational combinations that exploit synthetic lethality could expand efficacy, but they must be evaluated with caution to avoid compounding toxicities. At present, no PCNA inhibitor has received FDA approval, and all remain in early-stage clinical evaluation. The results of ongoing trials with next-generation candidates such as AOH1996 will determine whether this long-considered “undruggable” protein can be realized as a viable therapeutic target.

## 4 Targeting MDM2 for cancer therapy: are we there yet?

### 4.1 MDM2, a multifaceted oncogene: beyond p53

**FIGURE 4:**
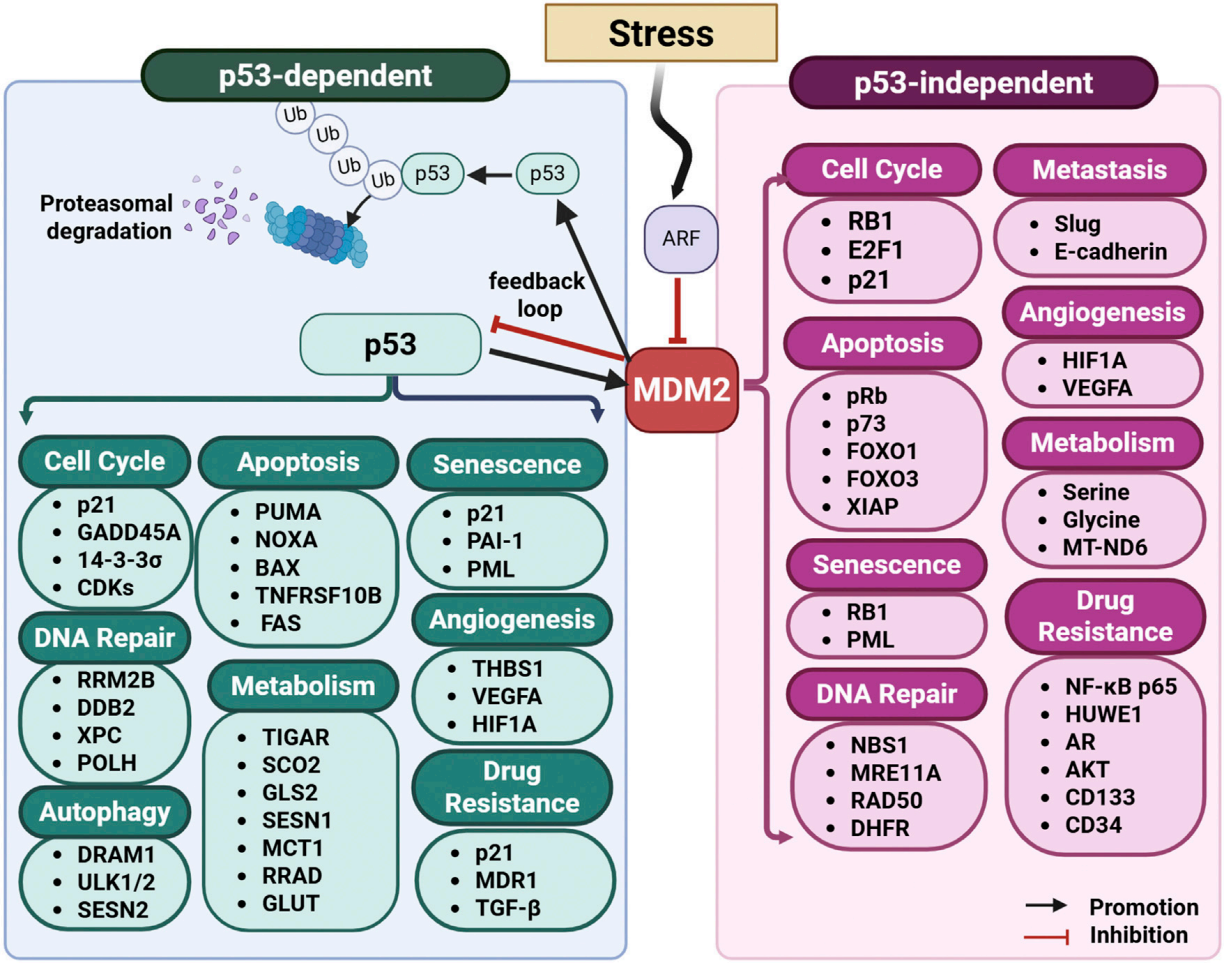
MDM2 pathway: p53-dependent and p53-independent programs. Schematic overview of MDM2’s central role in stress signaling and cell-fate control. DNA damage and other stresses activate checkpoint pathways and ARF, which suppresses MDM2 and stabilizes p53. Activated p53 transcriptionally induces MDM2, establishing a negative-feedback loop. MDM2 then ubiquitinates p53, targeting it for proteasomal degradation. MDM2 regulates modules that drive outcome-specific programs, including cell cycle, DNA repair, autophagy, apoptosis, angiogenesis, metabolism, and senescence, through both p53-dependent (left panel) and p53-independent (right panel) mechanisms. Abbreviations: 14-3-3σ (SFN): Stratifin; ABCB1 (MDR1): ATP-binding cassette subfamily B member 1; AKT: Protein kinase B; AR: Androgen receptor; BAX: BCL2-associated X protein; BBC3 (PUMA): BCL2-binding component 3; CD133 (PROM1/PROML1): Prominin-1; CD34: Cluster of differentiation 34; CDH1 (E-cadherin): Cadherin-1; CDKs: Cyclin-dependent kinases; CDKN1A (p21): Cyclin-dependent kinase inhibitor 1A; DDB2: DNA damage-binding protein 2; DHFR: Dihydrofolate reductase; DR5 (TNFRSF10B): Death receptor 5; DRAM1: DNA-damage regulated autophagy modulator 1; E2F1: E2F transcription factor 1; FAS (CD95): Fas cell-surface death receptor; FOXO1/FOXO3: Forkhead box O1/O3; GADD45A: Growth arrest and DNA-damage-inducible alpha; GLS2: Glutaminase 2; GLUT: Glucose transporter; glycine: the amino acid glycine; HIF1A: Hypoxia-inducible factor 1-alpha; HUWE1: HECT, UBA and WWE domain-containing E3 ubiquitin ligase 1; MCT1 (SLC16A1): Monocarboxylate transporter 1; MRE11A: MRE11 homolog A; MT-ND6: Mitochondrially encoded NADH dehydrogenase 6; NBN (NBS1): Nibrin; NF-κB p65 (RELA): Nuclear factor κB subunit p65; NOXA (PMAIP1): Phorbol-12-myristate-13-acetate–induced protein 1; PAI-1 (SERPINE1): Plasminogen activator inhibitor-1; PML: Promyelocytic leukemia protein; POLH: DNA polymerase eta; PROML1 (CD133): Prominin-1; pRb (RB1): Retinoblastoma protein; p73 (TP73): Tumor protein p73; RB1: Retinoblastoma 1; RAD50: RAD50 double-strand break repair protein; RELA: See NF-κB p65; RRAD: Ras-related associated with diabetes; RRM2B (p53R2): Ribonucleotide-diphosphate reductase subunit M2B; SCO2: Synthesis of cytochrome c oxidase 2; serine: the amino acid serine; SESN1/SESN2: Sestrin 1/2; SNAI2 (Slug): Snail family transcriptional repressor 2; THBS1 (TSP-1): Thrombospondin-1; TGF-β: Transforming growth factor-beta; TIGAR: TP53-induced glycolysis and apoptosis regulator; TNFRSF10B (DR5): Tumor necrosis factor receptor superfamily member 10B; TP73 (p73): Tumor protein p73; ULK1/ULK2: UNC-51–like kinase 1/2; Ub: ubiquitin; VEGFA: Vascular endothelial growth factor A; XIAP: X-linked inhibitor of apoptosis; XPC: Xeroderma pigmentosum group C.

Mouse Double Minute 2 (MDM2) is a multifaceted oncogene that plays a critical role in the regulation of cellular processes, particularly through its interaction with the tumor suppressor protein p53 (Figure 4) (Fakharzadeh et al., 1991; Honda et al., 1997; Levine, 2020; Momand et al., 1992; Oliner et al., 1992). Originally identified as an oncogene amplified in murine tumor cells, MDM2 has since emerged as a central player in tumorigenesis (Freedman et al., 1999). Its primary function is to regulate p53 levels by acting as an E3 ubiquitin ligase, tagging p53 for proteasomal degradation (Fakharzadeh et al., 1991; Honda et al., 1997; Levine, 2020; Momand et al., 1992; Oliner et al., 1992). This mechanism is a cornerstone of cellular homeostasis, as it ensures that p53 activity is tightly controlled under normal conditions.

Structurally, MDM2 is composed of several domains that mediate its diverse functions (Nag et al., 2013; Nag et al., 2014). The N-terminal domain contains a hydrophobic pocket that binds the transactivation domain of p53, effectively blocking its transcriptional activity (Chi et al., 2005; Kussie et al., 1996; Nag et al., 2013). This domain is the primary target for small-molecule inhibitors aimed at disrupting the MDM2-p53 interaction. The central acidic domain interacts with various proteins and contributes to MDM2’s ability to regulate chromatin dynamics and transcription (Nag et al., 2013; Nag et al., 2014). The C-terminal RING finger domain, which mediates E3 ubiquitin ligase activity, is essential for p53 ubiquitination and also facilitates MDM2’s autoubiquitination, a process that regulates its own degradation (Fang et al., 2000; Iwakuma and Lozano, 2003).

MDM2’s oncogenic role extends beyond its regulation of p53, as it also participates in p53-independent pathways (Figure 4) (Nag et al., 2013; Nag et al., 2014; Wang W. et al., 2024). It interacts with numerous proteins, including transcription factors (e.g., E2F1 and NF-κB), signaling molecules, and components of the chromatin remodeling machinery (Nag et al., 2013; Nag et al., 2014; Wang W. et al., 2024). Through these interactions, MDM2 influences processes such as cell cycle progression, differentiation, and DNA repair (Wang W. et al., 2024). MDM2 has been shown to drive genomic instability through its interaction with Nijmegen breakage syndrome protein 1 (Nbs1), a component of the MRN complex (Mre11-Rad50-Nbs1), delaying DNA repair and promoting tumorigenesis (Alt et al., 2005). Additionally, MDM2 contributes to stemness maintenance by engaging with Polycomb repressor complexes (PRC1 and PRC2), facilitating epigenetic modifications that sustain an undifferentiated cellular state (Wen et al., 2014; Wienken et al., 2016). In cancer models, MDM2 depletion enhances osteoblastic differentiation and reduces the efficiency of induced pluripotent stem cell (iPSC) formation, further highlighting its role in stem cell maintenance (Wienken et al., 2016). Beyond its well-documented role in p53 degradation, MDM2 ubiquitinates and regulates multiple oncogenic and tumor-suppressor proteins, impacting cancer proliferation, survival, and drug resistance. It serves as an E3 ligase for histone deacetylase 3 (HDAC3), forkhead box O4 (FOXO4), and insulin-like growth factor 1 receptor (IGF-1R), affecting cellular migration, metabolic adaptation, and tumor growth (Brenkman et al., 2008; Choi et al., 2019; Girnita et al., 2003). Additionally, MDM2 stabilizes oncogenic factors such as E2F1 and STAT5 by preventing their degradation, reinforcing cancer cell survival (Zhang et al., 2005; Zhou J. et al., 2021).

Emerging studies have identified MDM2 as a key player in cancer metabolism, influencing glycolysis, oxidative stress responses, and mitochondrial function (Wang W. et al., 2024). It modulates NAD+/NADH balance, affecting glutathione recycling, and supports serine and glycine metabolism, which is critical for nucleotide biosynthesis in tumor cells (Cissé et al., 2020; Riscal et al., 2016). Under hypoxic and oxidative stress conditions, MDM2 is imported into mitochondria, where it inhibits NADH-dehydrogenase 6 (MT-ND6), leading to increased reactive oxygen species (ROS) production and mitochondrial dysfunction (Arena et al., 2018; Elkholi et al., 2019). MDM2 also modulates the immune response within the tumor microenvironment, impacting T cell function, cytokine signaling, and antigen presentation. It has been identified as a tumor-associated antigen (TAA) in chronic lymphocytic leukemia (CLL), making it an attractive target for T cell-based immunotherapies (Mayr et al., 2006). Additionally, MDM2 interacts with STAT5, preventing its degradation and stabilizing its expression in tumor-infiltrating T cells, further contributing to tumor progression (Zhou J. et al., 2021). Pharmacological inhibition of MDM2 enhances dendritic cell activation, increases CD8^+^ T cell cytotoxicity, and shifts the CD8+/Treg balance to favor anti-tumor immunity (Wang HQ. et al., 2021).

MDM2 plays a pivotal role in cellular regulation, primarily through its control of p53, and its dysregulation contributes significantly to tumorigenesis. Under normal conditions, MDM2 function is tightly regulated by multiple upstream pathways. The ADP-ribosylation factor (ARF) tumor suppressor inhibits MDM2 during oncogenic stress, stabilizing p53 and promoting apoptosis (Nag et al., 2013; Nag et al., 2014). Additionally, DNA damage activates kinases such as ATM and ATR, which phosphorylate MDM2 and disrupt its interaction with p53, ensuring appropriate cellular responses to genomic stress (Nag et al., 2013; Nag et al., 2014). However, MDM2 amplification or overexpression can override these regulatory mechanisms, leading to p53 inactivation, uncontrolled proliferation, and tumor progression. This highlights MDM2’s dual role as both a key regulator of cellular homeostasis and a driver of malignancy.

Clinically, MDM2 is frequently dysregulated in various cancers, where its amplification (Table 6) or overexpression serves as a key oncogenic event. It is commonly observed in malignancies such as sarcomas, glioblastomas, breast cancers, lung cancers, and hematologic cancers (Nag et al., 2013; Nag et al., 2014; Oliner et al., 2016; Wang W. et al., 2024). In tumors that retain wild-type p53, MDM2 amplification provides an alternative mechanism for evading apoptosis and sustaining unchecked proliferation. Its overexpression is particularly prevalent in soft tissue sarcomas, such as well-differentiated and dedifferentiated liposarcomas, where it serves as a diagnostic marker and is associated with aggressive disease and poor prognosis (Sun and Crago, 2023). Similarly, glioblastoma with high MDM2 levels exhibits greater resistance to therapy and reduced survival outcomes (Sato et al., 2011), while breast cancer frequently displays elevated MDM2 expression, correlating with poor clinical progression (Turbin et al., 2006; Yao et al., 2024). Given its widespread involvement in tumor development and progression, MDM2 is also recognized as a prognostic biomarker, with high expression levels linked to advanced disease stages and treatment resistance. For instance, in sarcomas and glioblastomas, MDM2 amplification correlates with poor prognosis, while in breast cancer, elevated MDM2 levels are associated with reduced disease-free survival, particularly in estrogen receptor-positive tumors (Oliner et al., 2016). These findings emphasize the utility of MDM2 as a biomarker for patient stratification and targeted therapeutic approaches.

**TABLE 6:** Pan-cancer frequency of MDM2 genomic alterations.

Cancer types	Total rate (%)	Alteration (%)			
		Mutation	Amplication	Structural variant	Multiple alterations
Soft Tissue Sarcoma	20.62	0.11	19.71	0.00	0.80
Lung Cancer	16.37	1.82	14.55	0.00	0.00
Nerve Sheath Tumor	9.52	0.00	9.52	0.00	0.00
Ampullary Cancer	9.09	0.00	9.09	0.00	0.00
Bladder Cancer	7.95	0.71	7.19	0.00	0.05
Penile Cancer	7.69	7.69	0.00	0.00	0.00
Sex Cord Stromal Tumor	7.69	0.00	7.69	0.00	0.00
Non Small Cell Lung Cancer	6.33	0.69	5.54	0.00	0.10
Urothelial Carcinoma	6.25	1.04	5.21	0.00	0.00
Gallbladder Carcinoma	6.25	0.00	6.25	0.00	0.00
Bone Sarcoma	6.01	0.00	6.01	0.00	0.00
Skin Cancer, Non-Melanoma	5.77	5.77	0.00	0.00	0.00
Non-Small Cell Lung Cancer	5.68	0.61	4.98	0.03	0.06
Extrahepatic Cholangiocarcinoma	5.41	0.00	5.41	0.00	0.00
Bone Cancer	5.39	0.41	4.98	0.00	0.00
Germ Cell Tumor	5.39	0.31	5.08	0.00	0.00
Intrahepatic Cholangiocarcinoma	5.22	0.54	4.68	0.00	0.00

Data sources from cBioPortal.org (date to 11 Sep 2025). Total cases were more than 10 and MDM2 gene altered frequencies above 5% are listed.

MDM2 overexpression has been linked to resistance against chemotherapy, radiotherapy, tyrosine kinase inhibitors (TKIs), and immune checkpoint inhibitors (ICIs) (Wang W. et al., 2024). MDM2 suppresses p53 and contributes to drug resistance in glioblastomas, pancreatic, breast, and gastric cancers by regulating factors such as O-6-methylguanine-DNA methyltransferase (MGMT), Musashi-2, and homeobox A13 (HOXA13) (Han et al., 2018; Sato et al., 2011; Sheng et al., 2017). Additionally, it enhances EMT and stem cell properties, further increasing treatment resistance (Sun and Tang, 2016). Studies have demonstrated that MDM2 inhibition sensitizes tumors to chemotherapy and radiotherapy by restoring p53-dependent apoptosis (Ringshausen et al., 2006; Werner et al., 2015; Yi et al., 2018). MDM2 amplification is also associated with TKI resistance, particularly in lung cancer, where its inhibition has been explored as a means to overcome resistance to epidermal growth factor receptor (EGFR)-targeted therapies and BCR-ABL1 inhibitors in chronic myeloid leukemia (Carter et al., 2020; Dworakowska et al., 2004; Sun et al., 2020). Beyond conventional therapies, MDM2 has been implicated in immune checkpoint inhibitor (ICI) resistance and hyperprogressive disease (HPD). Its overexpression has been identified as a biomarker for HPD, with studies showing that MDM2 suppresses anti-tumor immunity by reducing T cell activation and promoting an immunosuppressive environment (Adashek et al., 2020). Preclinical studies indicate that MDM2 inhibition enhances immune responses by increasing T cell infiltration and inflammatory gene expression, suggesting its potential for combination strategies with ICIs (Wang HQ. et al., 2021; Zhou X. et al., 2021). Given its broad impact on therapy resistance, targeting MDM2, either alone or in combination with chemotherapy, TKIs, or immunotherapy, offers a promising strategy to improve treatment efficacy and patient outcomes.

Efforts to target MDM2 have primarily focused on disrupting its interaction with p53 to restore p53 activity and trigger tumor cell apoptosis. Such approaches have shown promise in preclinical studies and early-phase clinical trials, particularly in hematologic malignancies and solid tumors with MDM2 amplification. However, challenges remain in optimizing these therapies to maximize efficacy while minimizing toxicity.

### 4.2 Targeting MDM2: challenges, failures, and breakthroughs

**FIGURE 5:**
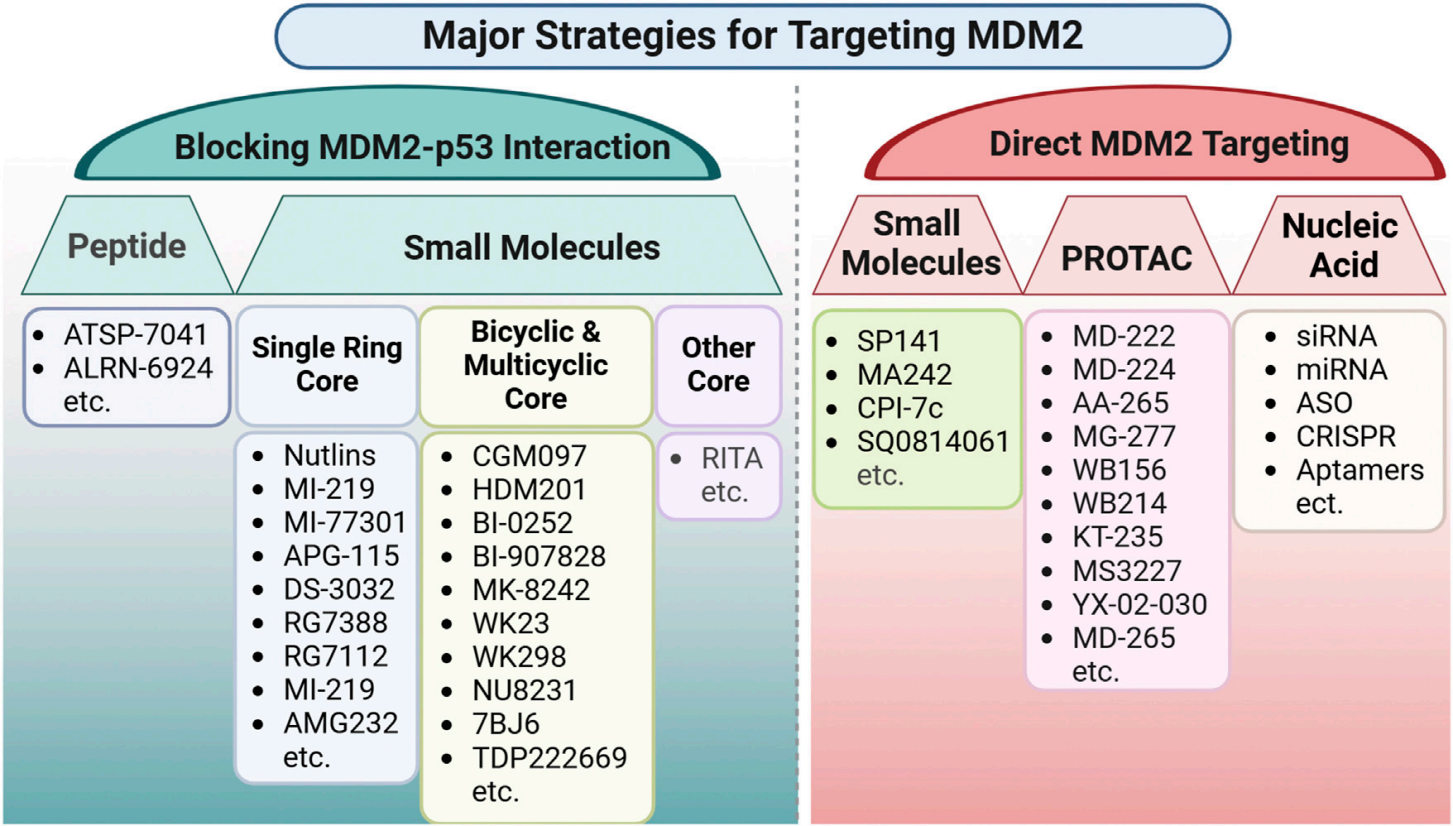
Comparison strategies targeting MDM2-p53 binding and direct MDM2 inhibitors and degraders. Schematic summary of therapeutic approaches grouped by mechanism. Left panel: Blocking the MDM2–p53 interaction. Agents that occupy the p53-binding pocket on MDM2 to restore p53 activity, including peptides and small-molecule scaffolds (single-ring, bicyclic/multicyclic, and other cores). Right panel: Direct MDM2 targeting. Modalities that inhibit or eliminate MDM2 independently of p53 binding, including small molecules, PROTAC degraders, and nucleic-acid strategies (siRNA/miRNA, antisense oligonucleotides, CRISPR guides, and aptamers). Abbreviations: PROTAC, proteolysis-targeting chimera; siRNA, small interfering RNA; miRNA, microRNA; CRISPR, clustered regularly interspaced short palindromic repeats; ASO, antisense oligonucleotide.

Over the past few decades, significant efforts have been made to develop strategies targeting MDM2, given its central role in tumorigenesis and therapy resistance. Approaches range from peptide-based inhibitors and antisense oligonucleotides to small molecules designed to disrupt the MDM2-p53 interaction or directly degrade MDM2 [reviewed in (Beloglazkina et al., 2020; Fang et al., 2020b; Hu et al., 2025; Liu et al., 2019; Twarda-Clapa, 2024; Wang W. et al., 2024; Yao et al., 2024)]. Figure 5 compares strategies targeting p53-MDM2 binding with direct MDM2 inhibitors and degraders.

The initial small-molecule inhibitors focused on blocking MDM2’s binding to p53, preventing p53 degradation and restoring its tumor-suppressive functions. The discovery of the crystal structure of the MDM2-p53 complex (Kussie et al., 1996) provided a structural basis for the design of inhibitors that mimic p53’s interaction motifs, leading to the development of various small-molecule inhibitors, including Nutlins, spiro-oxindoles, pyrrolidone derivatives, and bicyclic core compounds (Beloglazkina et al., 2020; Fang et al., 2020b; Liu et al., 2019). These inhibitors demonstrated therapeutic potential in preclinical models, but their clinical development has been limited by issues related to toxicity, resistance, and poor bioavailability (Abdul Razak et al., 2022; Daver et al., 2023; Gounder et al., 2023; Konopleva et al., 2022; Mascarenhas et al., 2022; Moschos et al., 2022; Sekiguchi et al., 2023).

Among the most studied inhibitors, peptide-based agents were initially developed to mimic the α-helical domain of p53 and disrupt its interaction with MDM2 (Garcia-Echeverria et al., 2000). Despite advances, these peptides exhibited low binding affinity due to conformational differences between peptides and full-length p53 (Sakurai et al., 2004). More stable cyclic peptides, such as ATSP-7041 and its optimized version ALRN-6924, demonstrated improved efficacy by inhibiting both MDM2 and MDMX, thereby enhancing p53 stabilization (Carvajal et al., 2018; Chang et al., 2013). Non-peptide small-molecule inhibitors like Nutlins, which mimic key p53 residues (Phe19, Trp23, Leu26), emerged as promising alternatives. Nutlin-3a, a potent cis-imidazoline derivative, was the first to establish proof-of-concept for MDM2 inhibition (Tovar et al., 2006; Vassilev et al., 2004). Despite showing activity in preclinical models, Nutlins had limited clinical utility due to pharmacokinetic challenges. Further refinements led to second-generation inhibitors such as RG7112 and RG7388 (Idasanutlin), which displayed improved bioavailability and selectivity but encountered dose-limiting toxicities in clinical trials (Vu et al., 2013). Spiro-oxindole inhibitors, including MI-77301 (SAR405838) and its optimized analog APG-115, were developed to enhance binding affinity and stability (Aguilar et al., 2017; Wang S. et al., 2014). Another class, represented by AMG232 (Navtemadlin) and BI 907828, utilized distinct structural scaffolds to improve pharmacokinetic properties and efficacy (Gessier et al., 2015; Rew and Sun, 2014). However, challenges such as resistance mechanisms and toxicity remain barriers to widespread clinical application.

Beyond targeting the MDM2-p53 interaction, recent efforts have focused on small molecules that directly modulate MDM2 expression or activity. Since MDM2 functions beyond p53 regulation, strategies that reduce its expression, inhibit its enzymatic activity, or induce its degradation may offer broader therapeutic benefits. Several small-molecule inhibitors, including β-carboline-based chalcones like CPI-7c (Singh et al., 2016), have demonstrated potential in downregulating MDM2, expanding the scope of direct MDM2 inhibitors. Additionally, makaluvamine analogs (Wang et al., 2019b; Wang et al., 2018; Wang et al., 2009) and SP141 (Cissé et al., 2020; Patil et al., 2017; Punganuru et al., 2020; Wang et al., 2019c; Wang et al., 2014b; Wang et al., 2014c; Wang et al., 2020) have been developed to promote MDM2 degradation, demonstrating strong anti-tumor effects independent of p53 status across multiple cancer types. These findings underscore a pan-cancer approach for targeting MDM2, broadening its therapeutic relevance beyond tumors harboring wild-type p53.

Recently, PROTAC-based approaches have gained traction, utilizing PROTACs to induce MDM2 degradation. Pioneering studies developed PROTACs like MD-222 and MD-224, which recruit cereblon E3 ligase to facilitate MDM2 degradation, significantly enhancing anti-tumor activity compared to conventional inhibitors (Li et al., 2019). WB156 and WB214 demonstrated remarkable potency by degrading both MDM2 and p53, with WB214 also acting as a molecular glue to degrade G1 to S phase transition 1 (GSPT1) (Wang B. et al., 2021; Wang B. et al., 2019). Another notable PROTAC, KT-253, has shown exceptional preclinical efficacy, triggering rapid apoptosis and sustained tumor regression in acute myeloid leukemia (AML) models, leading to its current investigation in Phase I clinical trials (Chutake et al., 2022). The FDA recently granted orphan drug designation to KT-253 for the treatment of AML, further highlighting its therapeutic potential. More recently, YX-02–030 (derived from RG7112) became the first MDM2-targeted PROTAC to demonstrate anticancer activity against p53 mutant cells in triple-negative breast cancer (TNBC) (Adams et al., 2023). In addition, MD-265 is a potent PROTAC MDM2 degrader that selectively depletes MDM2, activates p53 in wild-type p53 cancer cells, induces sustained tumor regression in leukemia models with minimal toxicity, and exhibits a favorable pharmacokinetic profile, making it a strong candidate for advanced preclinical cancer therapy development (Aguilar et al., 2024). AS1411-VH032 and homoAS1411 are novel tumor-targeting PROTACs that exploit AS1411 aptamer’s affinity for nucleolin to selectively degrade MDM2 in tumor cells, achieve effective tumor suppression with minimal toxicity, and offer a promising approach for precise and safe MDM2-targeted cancer therapy (Wang Z. et al., 2024). Despite their promise, PROTACs face challenges such as off-target toxicity, high molecular weight, poor solubility, and unfavorable pharmacokinetic properties, highlighting the need for further optimization to enhance clinical applicability (Chen et al., 2023; Edmondson et al., 2019).

The development of dual MDM2/MDMX inhibitors has emerged as another promising avenue. Since MDMX also binds and inhibits p53, targeting both MDM2 and MDMX may provide superior anti-tumor effects. Small-molecule inhibitors like MEL23 and MEL24 block the E3 ligase activity of the MDM2-MDMX complex, stabilizing p53 and promoting apoptosis (Herman et al., 2011). Other inhibitors, such as RO-2443 and RO-5963, were optimized for dual targeting, with RO-5963 exhibiting a 400-fold greater selectivity for MDMX inhibition compared to Nutlin-3a (Graves et al., 2012). Peptide-based inhibitors, including ALRN-6924 and ATSP-7041, also demonstrated dual inhibitory potential (Carvajal et al., 2018; Chang et al., 2013). Expanding beyond MDM2/MDMX, multi-targeted inhibitors such as MA242, which targets both MDM2 and NFAT1 (Wang et al., 2019b; Wang et al., 2018), and dual Bcl-2/MDM2 inhibitors (Pan et al., 2017), represent next-generation approaches to overcome resistance and improve efficacy.

Numerous MDM2 inhibitors from various pharmaceutical companies have been evaluated in clinical trials to assess their pharmacokinetics (PK), pharmacodynamics (PD), and safety and efficacy (alone or in combination). However, several have faced challenges, leading to trial terminations or clinical holds. Our previous review provided a comprehensive analysis of the clinical landscape of MDM2 inhibitors (Wang W. et al., 2024). In this review, we have updated the status of ongoing and actively recruiting clinical trials, reflecting the latest developments in the field (Table 7). Several MDM2 inhibitors are currently undergoing Phase III clinical evaluation. Notably, idasanutlin (RG7388) was assessed in the Phase III MIRROS trial (NCT02545283) in combination with cytarabine for relapsed or refractory acute myeloid leukemia (AML), but the study failed to meet its primary endpoint, as the addition of idasanutlin did not improve overall survival compared to cytarabine alone (median, 8.3 vs. 9.1 months) (Konopleva et al., 2022). Similarly, milademetan (RAIN-32) was evaluated in the Phase III MANTRA trial (NCT04979442) for dedifferentiated liposarcoma, but it did not demonstrate a significant improvement in progression-free survival compared to trabectedin (Gounder, 2022). Despite these setbacks, MDM2 inhibitors such as navtemadlin and brigimadlin continue to advance in clinical development, with multiple ongoing Phase II and III trials investigating their efficacy in malignancies, including myelofibrosis and glioblastoma. These ongoing studies highlight the sustained interest in MDM2 as a therapeutic target in oncology, underscoring the need for continued refinement and novel approaches to enhance the clinical success of MDM2-targeting therapies (Table 7).

**TABLE 7:** Updated Summary of recruiting and active MDM2 Inhibitor Clinical Trials.

MDM2 inhibitor	Combination	Sponsor	Study title	Conditions	Objective	Clinical trails	NCT number
Status: not yet recruiting							
APG-115	MEK Inhibitor: Selumetinib	AeRang Kim	Early Phase Study Evaluating MEK and MDM2 Inhibition in Patients With NF1 and MPNST	Atypical Neurofibroma, Malignant Peripheral Nerve Sheath Tumor (MPNST), Neurofibromatosis 1 (NF1)	Safety, Tolerability, Pharmacokinetics (PK), Recommended Doses	Phase 0/I/II	NCT06735820
APG-115		National Cancer Institute (NCI)	Alrizomadlin (APG-115) in Subjects With BAP1 Cancer Syndrome and Early-Stage Mesothelioma	BRCA1-Associated Protein-1 (BAP1) Mutations, Early-stage BAP1-associated Malignancies, Early-stage Mesothelioma, Malignant Mesothelioma	Efficacy	Phase II	NCT06654050
Brigimadlin (BI 907828)	PD1 Inhibitor: Ezabenlimab	Institut Bergonié	Targeting MDMD and PD1 in Tumors With Tertiary Lymphoid Structures (EMPIRE)	Adult Soft Tissue Sarcoma, Non Small Cell Lung Cancer, Triple Negative Breast Cancer, Colorectal Cancer, Biliary Tract Cancer	Safety, Efficacy	Phase II	NCT06084689
SA53-OS		Lamassu Bio	Safety and Preliminary Efficacy of SA53-OS in Patients With Locally Advanced or Metastatic Solid Tumors	p53 Wild-type Refractory Solid Tumors	Safety, Efficacy, PK	Phase I/IIa	NCT06578624
Status: Recruiting							
APG-115	Azacitidine or Cytarabine	Ascentage Pharma	A Phase Ib Study of APG-115 Single Agent or in Combination With Azacitidine or Cytarabine in Patients With AML and MDS.	Acute Myeloid Leukemia (AML), Myelodysplastic Syndromes (MDS)	Safety, PK/Pharmacodynamic (PD), Maximum Tolerated Dose (MTD)/Recommended Phase 2 Dose (RP2D)	Phase Ib	NCT04275518
APG-115	PD1 Inhibitor: Pembrolizumab	Ascentage Pharma	A Study of APG-115 in as a Monotherapy or Combination With Pembrolizumab in Patients With Metastatic Melanomas or Advanced Solid Tumors	MDM2 Gene Mutation, Cutaneous Melanoma, MPNST, Melanoma, Mucosal Melanoma, p53 Mutation, Unresectable or Metastatic Melanoma or Advanced Solid Tumors, Uveal Melanoma	Safety, Tolerability	Phase I/II	NCT03611868
APG-115	BCL-2 Inhibitor: APG-2575	Ascentage Pharma	APG-115 Alone or in Combination With APG-2575 in Children With Recurrent or Refractory Neuroblastoma or Solid Tumors	Recurrent or Refractory Neuroblastoma, Solid Tumor	MTD, RP2D, Safety, PK, Initial Efficacy	Phase I	NCT05701306
APG-115	PD-1 Inhibitor: Toripalimab	Ascentage Pharma	APG-115 in Combination With PD-1 Inhibitor in Patients With Advanced Liposarcoma or Advanced Solid Tumors	Advanced Solid Tumor, Liposarcoma	MTD, RP2D, Safety, Efficacy	Phase Ib/II	NCT04785196
APG-115	BCL-2 Inhibitor: APG-2575	Ascentage Pharma	A Study Evaluating APG-115 as a Single Agent or in Combination With APG-2575 in Subjects With R/​R T-PLL and NHL	Non-Hodgkins Lymphoma, T-Prolymphocytic Leukemia	PK, Safety, Efficacy	Phase IIa	NCT04496349
APG-115	Azacitidine	Ascentage Pharma	A Study of APG-115 Alone or Combined With Azacitidine in Patients With AML, CMML, or MDS	AML, Chronic Myelomonocytic Leukemia (CMML)	Dose Escalation	Phase Ib/II	NCT04358393
Navtemadlin (KRT-232)	Tyrosine Kinase Inhibitor (TKI): Dasatinib or Nilotinib	Kartos Therapeutics	KRT-232 and TKI Study in Chronic Myeloid Leukemia	Chronic Myeloid Leukemia	Safety, Efficacy	Phase I/IIa	NCT04835584
Navtemadlin (KRT-232)	JAK Inhibitor: TL-895	Kartos Therapeutics	KRT-232 in Combination With TL-895 for the Treatment of R/R MF and KRT-232 for the Treatment of JAKi Intolerant MF	Myelofibrosis, Post-Essential Thrombocythemia Myelofibrosis (Post-ET-MF), Post-Polycythemia Vera Myelofibrosis (Post-PV-MF), Primary Myelofibrosis (PMF)	Safety, Efficacy	Phase Ib/II	NCT04640532
Navtemadlin (KRT-232)		Kartos Therapeutics	Study of Navtemadlin as Maintenance Therapy in TP53WT Advanced or Recurrent Endometrial Cancer	TP53WT Advanced or Recurrent Endometrial Cancer	Safety, Efficacy	Phase II/III	NCT05797831
Navtemadlin (KRT-232)		Kartos Therapeutics	Study of KRT-232 or TL-895 in Janus Associated Kinase Inhibitor Treatment-NaÃ¯ve Myelofibrosis	Post-ET-MF, Post-PV-MF, PMF	Safety, Tolerability, Efficacy	Phase II	NCT04878003
Navtemadlin (KRT-232)	Best Available Therapy (BAT)	Kartos Therapeutics	KRT-232 Versus Best Available Therapy for the Treatment of Subjects With Myelofibrosis Who Are Relapsed or Refractory to JAK Inhibitor Treatment	Post-ET-MF, Post-PV-MF, PMF	Recommended Dose, Dosing Schedule	Phase II/III	NCT03662126
Navtemadlin (KRT-232)	Ruxolitinib	Kartos Therapeutics	Study of Navtemadlin Add-on to Ruxolitinib in JAK Inhibitor-NaÃ¯ve Patients with Myelofibrosis Who Have a Suboptimal Response to Ruxolitinib	MF, Post-PV MF, PMF	Safety, Efficacy	Phase III	NCT06479135
Navtemadlin (KRT-232)	Anti-PD-1/Anti-PD-L1: Avelumab	Kartos Therapeutics	Navtemadlin (KRT-232) With or Without Anti-PD-1/Anti-PD-L1 for the Treatment of Patients With Merkel Cell Carcinoma	Merkel Cell Carcinoma	Safety, Efficacy	Phase Ib/II	NCT03787602
HDM201	TKI: Pazopanib	Centre Leon Berard	HDM201 and Pazopanib in Patients With P53 Wild-type Advanced/​Metastatic Soft Tissue Sarcomas (AMPHISARC)	P53 Wild-type Advanced/Metastatic Soft Tissue Sarcomas	Safety	Phase I/II	NCT05180695
Status: Active, not recruiting							
Brigimadlin (BI 907828)		Boehringer Ingelheim	Brightline-2: A Study to Test Whether Brigimadlin (BI 907828) Helps People With Cancer in the Biliary Tract, Pancreas, Lung or Bladder	Pancreatic Neoplasms, Solid Tumors, Biliary Tract Cancer, Lung Neoplasms, Bladder Cancer	Safety, Efficacy	Phase IIa/IIb	NCT05512377
Brigimadlin (BI 907828)	OATP Inhibitor: Rifampicin; CYP3 Inhibitor: Itraconazole	Boehringer Ingelheim	A Study in People With Advanced Cancer to Test Whether the Amount of BI 907828 in the Blood is Influenced by Taking an OATP Inhibitor or a CYP3 Inhibitor	Solid Tumors	Potential Drug-drug Interaction	Phase I	NCT05372367
Brigimadlin (BI 907828)	Doxorubicin	Boehringer Ingelheim	Brightline-1: A Study to Compare Brigimadlin (BI 907828) With Doxorubicin in People With a Type of Cancer Called Dedifferentiated Liposarcoma	Dedifferentiated Liposarcoma	Safety, Efficacy	Phase II/III	NCT05218499
Brigimadlin (BI 907828)	Radiotherapy	Boehringer Ingelheim	A Study to Determine How BI 907828 (Brigimadlin) is Taken up in the Tumor (Phase 0) and to Determine the Highest Dose of BI 907828 (Brigimadlin) That Could be Tolerated (Phase 1a) in Combination With Radiation Therapy in People With a Brain Tumor Called Glioblastoma	Glioblastoma	PK, Dose Escalation	Phase 0/Ia	NCT05376800
Brigimadlin (BI 907828)		Boehringer Ingelheim	A Study to Test Long-term Treatment With Brigimadlin in People With Solid Tumours Who Took Part in a Previous Study With This Medicine	Solid Tumors	Long-term Safety	Phase II	NCT06619509
Brigimadlin (BI 907828)		Boehringer Ingelheim	Brightline-4: A Study to Test How Well Brigimadlin is Tolerated by People With a Type of Cancer Called Dedifferentiated Liposarcoma	Liposarcoma, Dedifferentiated	Safety, Efficacy	Phase III	NCT06058793
Brigimadlin (BI 907828)	Immune checkpoint inhibitor: BI 754091(ezabenlimab) or BI 754091+BI 754111	Boehringer Ingelheim	A Study in Patients With Different Types of Advanced Cancer (Solid Tumors) to Test Different Doses of BI 907828 (Brigimadlin) in Combination With BI 754091 (Ezabenlimab) and BI 754111 or BI 907828 (Brigimadlin) in Combination With BI 754091 (Ezabenlimab)	Neoplasms	Dose Escalation	Phase Ia/Ib	NCT03964233
Brigimadlin (BI 907828)		Boehringer Ingelheim	This Study Aims to Find the Best Dose of BI 907828 (Brigimadlin) in Patients With Different Types of Advanced Cancer (Solid Tumors)	Advanced or Metastatic Solid Tumors	Dose Escalation	Phase Ia/Ib	NCT03449381
Navtemadlin (KRT-232)	Decitabine, BCL-2 Inhibitor: Venetoclax	National Cancer Institute (NCI)	Testing a New Chemotherapy Drug, KRT-232 (AMG-232) in Combination With Decitabine and Venetoclax in Patients With Acute Myeloid Leukemia	Recurrent, Refractory, Secondary Acute Myeloid Leukemia	Side Effects, Best Dose	Phase Ib	NCT03041688
Navtemadlin (KRT-232)	TKI: TL-895	Telios Pharma, Inc	TL-895 and KRT-232 Study in Acute Myeloid Leukemia	AML	Safety, Efficacy	Phase Ib/II	NCT04669067
Navtemadlin (KRT-232)	Cytarabine, Idarubicin Hydrochloride	NCI	Testing the Addition of an Anti-cancer Drug, Navtemadlin, to the Usual Treatments (Cytarabine and Idarubicin) in Patients With Acute Myeloid Leukemia	AML, AML Arising From Previous Myelodysplastic Syndrome	Toxicity, MTD, RP2D, Efficacy	Phase Ib	NCT04190550
Navtemadlin (KRT-232)	Radiation Therapy	NCI	Testing the Ability of AMG 232 (KRT 232) to Get Into the Tumor in Patients With Brain Cancer	Glioblastoma, MGMT-Unmethylated Glioblastoma, Recurrent Glioblastoma	Concentration, Safety, Toxicity, PK/PD	Phase 0/I	NCT03107780
Navtemadlin (KRT-232)	Radiotherapy	NCI	Navtemadlin and Radiation Therapy in Treating Patients With Soft Tissue Sarcoma	Wild-Type P53 Resectable Soft Tissue Sarcoma, Soft Tissue Sarcoma	Safety, Tolerability, MTD/RP2D, PK, PD, Toxicity, Efficacy	Phase Ib	NCT03217266
ALRN-6924	Paclitaxel	M.D. Anderson Cancer Center	ALRN-6924 and Paclitaxel in Treating Patients With Advanced, Metastatic, or Unresectable Solid Tumors	Advanced, Metastatic, or Unresectable Solid Tumors	Dose-limiting Toxicities (DLT), MTD/RP2D	Phase Ib	NCT03725436
APG-115	Carboplatin	Ascentage Pharma	APG-115 in Salivary Gland Cancer Trial	Malignant Salivary Gland Cancer, Salivary Gland Cancer	Efficacy	Phase I/II	NCT03781986

Abbreviations: AML, acute myeloid leukemia; BAP1, BRCA1-Associated Protein-1; BAT, best available therapy; BcL-2, B-cell lymphoma 2; BRCA1, BReast CAncer gene 1; CMML, chronic myelomonocytic leukemia; CYP3A, Cytochrome P450 3A; JAK, janus kinase; MDS, myelodysplastic syndromes; MPNST, malignant peripheral nerve sheath tumor; MTD, maximum tolerated dose; MEK, Mitogen-activated protein kinase; MGMT, O^6^-methylguanine-DNA; methyltransferase; NCI, national cancer institute; NF1, Neurofibromatosis 1; OATP, organic anion transporting polypeptide; PD, pharmacodynamic; PD-1, Programmed cell death protein 1; PK, pharmacokinetics; PMF, primary myelofibrosis; Post-ET-MF, Post-Essential Thrombocythemia Myelofibrosis; Post-PV-MF, Post-Polycythemia Vera Myelofibrosis; RP2D, Recommended Phase 2 Dose; TKI, tyrosine kinase inhibitor.

The therapeutic targeting of the MDM2–p53 interaction represents one of the most compelling yet frustrating narratives in oncology drug development. Despite robust preclinical validation and extensive clinical testing across multiple agents, no MDM2 inhibitor has achieved regulatory approval (Wang W. et al., 2024). The central obstacle has been mechanism-based toxicity, with overwhelming clinical evidence demonstrating that hematologic and gastrointestinal adverse events consistently prevent adequate dose escalation (Pi et al., 2019; Wang W. et al., 2024). This reflects the unavoidable on-target, off-tumor activation of wild-type p53 in normal tissues with high proliferative turnover, particularly hematopoietic stem cells and gastrointestinal epithelium, which results in an irreducibly narrow therapeutic window (Wang W. et al., 2024). Among adverse effects, hematologic toxicities are the most consistent and clinically limiting, with cytopenias, especially thrombocytopenia, emerging as the hallmark dose-limiting toxicity. Additional reported DLTs include gastrointestinal events, metabolic disturbances, fatigue, and cardiovascular toxicity, although these are generally less consistent across agents (Pi et al., 2019; Wang W. et al., 2024). Clinical experience across first-generation MDM2 antagonists highlights this recurrent challenge. RG7112, the first-in-class Nutlin derivative, confirmed target engagement through induction of p53 and its downstream effectors, yet development was halted because of poor tolerability and severe hematologic and gastrointestinal toxicities at clinically required doses (Andreeff et al., 2016). Idasanutlin (RG7388), developed as a more potent successor with improved pharmacokinetics, also failed to overcome these barriers. When combined with venetoclax in patients with relapsed or refractory AML, febrile neutropenia occurred in nearly half of participants, forcing dose modifications in most cases and contributing to early termination of the phase III MIRROS trial (Daver et al., 2023). AMG232 (KRT-232) was similarly constrained, with maximum tolerated doses determined to be substantially lower than predicted efficacious levels because of persistent hematological cytopenias and gastrointestinal intolerance (Gluck et al., 2020). The development of milademetan further illustrates these toxicity barriers. Although an intermittent schedule of 260 mg on days 1–3 and 15 to 17 every 28 days reduced hematological adverse events relative to continuous dosing, significant gastrointestinal toxicity persisted, and an adequate therapeutic window was not achieved, preventing regulatory progress (Gounder et al., 2023). Other optimized spirooxindole derivatives, including HDM201 and BI 907828, continued to display the class-typical profile of delayed-onset thrombocytopenia and gastrointestinal toxicities despite improved potency and pharmacokinetics (LoRusso et al., 2023; Stein et al., 2022). Beyond small molecules, ALRN-6924 (Aileron), a stapled peptide, demonstrated manageable safety profiles in Phase I/II trials with recommended dosing at 3.1 mg/kg (Saleh et al., 2021). However, hematologic toxicities persisted in combination settings, and development in NSCLC was discontinued after failing to demonstrate sufficient hematoprotection in chemotherapy regimens (Saleh et al., 2021). The collective experience across more than a dozen MDM2 inhibitors demonstrates that the therapeutic index of this drug class remains insufficient for viable clinical application. Nearly 2 decades of development and clinical testing across hematologic and solid malignancies have consistently shown that toxicity in normal tissues prevents dose intensification, leaving the promise of MDM2 inhibition unrealized in practice.

Looking ahead, innovative strategies are reshaping MDM2-targeted therapies, addressing key challenges in selectivity, efficacy, and resistance. Beyond traditional inhibitors, emerging approaches such as PROTACs, dual MDM2/MDMX inhibitors, and direct MDM2 degraders are broadening therapeutic potential, particularly in tumors with p53 mutations or non-p53-dependent pathways. Biomarker-driven patient stratification will be crucial, as MDM2’s diverse roles extend beyond p53 regulation. Combination therapies with chemotherapy, TKIs, or immune checkpoint inhibitors may overcome resistance and enhance treatment responses. Advances in nanotechnology-based drug delivery can optimize solubility, stability, and tumor targeting, while nucleic acid therapeutics, including siRNA, CRISPR, and aptamers, offer precision in modulating MDM2 activity. As research uncovers new MDM2 functions, integrating these strategies will be key to maximizing therapeutic success and improving clinical outcomes in oncology.

### 4.3 Translational advances and clinical barriers in MDM2 inhibition

The clinical development of MDM2 inhibitors represents a paradigmatic case of successful target validation coupled with formidable translational challenges. While preclinical models consistently demonstrated potent anti-tumor effects through p53 reactivation, clinical translation has been hampered by a fundamental biological constraint, most notably toxicity and the emergence of drug resistance. This section examines the key factors governing the clinical application of MDM2-targeted therapies, focusing on patient selection, contextual determinants of response, and strategies to overcome therapeutic limitations.

#### 4.3.1 Pan-cancer applicability

The overexpression or amplification of the MDM2 oncogene is a recurrent event across a broad spectrum of human cancers, including carcinomas of the lung, breast, liver, and gastrointestinal tract, as well as a defining feature of malignancies like well-differentiated and dedifferentiated liposarcoma (Wade et al., 2013; Wang W. et al., 2024). Clinically, this dysregulation is consistently associated with aggressive disease progression, therapy resistance, and poor patient outcomes, solidifying MDM2’s role as a critical pan-cancer biomarker and therapeutic target (Momand et al., 1998; Onel and Cordon-Cardo, 2004; Wang W. et al., 2024; Ware et al., 2014). The applicability of targeting MDM2 extends beyond tumors with wild-type p53, as it maintains significant oncogenic potency in p53-deficient cancers through alternative signaling pathways (Nag et al., 2013; Nag et al., 2014; Wang W. et al., 2024). This dual functionality establishes MDM2 inhibition as a versatile therapeutic strategy with broad relevance across diverse cancer types, driving ongoing research into novel inhibitors and rational combination therapies.

#### 4.3.2 Cancer-specific considerations

Response patterns to MDM2 inhibition vary significantly across cancer types. Early clinical signals were observed in hematologic cancers, particularly acute myeloid leukemia (AML), where the proliferative nature of hematopoietic cells and their reliance on MDM2–p53 regulation suggested heightened sensitivity (Daver et al., 2023). However, despite encouraging preclinical data, the phase III MIRROS trial of idasanutlin plus cytarabine in relapsed/refractory AML failed to improve survival, with efficacy limited by dose-limiting myelosuppression and rapid emergence of resistance (Konopleva et al., 2022). In solid tumors, responses have generally been modest, with the most compelling benefit seen in well-/dedifferentiated liposarcoma (WD/DD-LPS), where MDM2 amplification on chromosome 12q15 is nearly universal and frequently co-occurs with CDK4 amplification (Traweek et al., 2022). This genomic hallmark has established liposarcoma as the lead clinical setting for MDM2-directed therapy (Ray-Coquard et al., 2012). In contrast, solid tumors have demonstrated more modest response rates, with liposarcomas showing the greatest benefit among solid malignancies (Gounder, 2022). Importantly, tumors with TP53 mutations are intrinsically resistant to MDM2-p53 disruption therapies, necessitating careful genetic stratification (Nag et al., 2013; Nag et al., 2014; Wang W. et al., 2024).

#### 4.3.3 Key biomarkers for patient selection

Accurate patient selection is critical for MDM2-targeted therapy. The effective clinical application of MDM2 inhibitors relies heavily on robust biomarkers for patient selection, with wild-type TP53 status representing the paramount and non-negotiable prerequisite for efficacy with first-generation inhibitors that function by disrupting the MDM2-p53 interaction. MDM2 gene amplification serves as the strongest predictive biomarker, defining a patient population with demonstrated susceptibility to MDM2 inhibition, particularly in sarcomas such as dedifferentiated liposarcoma, where near-universal amplification occurs (Ray-Coquard et al., 2012). Recent evidence from studies of milademetan in intimal sarcoma suggests that cyclin-dependent kinase inhibitor 2A (CDKN2A) loss and amplified twist-related protein 1 (TWIST1) may serve as additional biomarkers associated with enhanced anti-tumor activity in MDM2-amplified tumors (Koyama et al., 2023). Importantly, clinical observations suggest that MDM2/MDM4 amplification may predict hyperprogressive disease under PD-1/PD-L1 blockade, emphasizing the need for cautious therapeutic sequencing in immuno-oncology (Fang W. et al., 2020; Kato et al., 2017).

#### 4.3.4 Contextual dependencies/tumor microenvironment

The therapeutic response to MDM2 inhibition is strongly influenced by the tumor microenvironment, encompassing immune regulation, metabolic adaptation, and stromal-immune crosstalk (Wang W. et al., 2024). Activation of the p53 pathway enhances tumor immunogenicity by upregulating antigen presentation machinery (e.g., MHC class II), inducing interferon signaling, and promoting IL-15 expression and T-cell infiltration (Zhao et al., 2025). At the same time, p53 activation can modulate PD-L1 expression at both transcriptional and post-translational levels, reshaping immune checkpoint dynamics in ways that may either potentiate or blunt anti-tumor immunity (Zhang et al., 2025). Clinically, MDM2-amplified tumors have been associated with hyperprogressive disease under PD-1/PD-L1 blockade, underscoring the need for rational sequencing and combination approaches (Fang W. et al., 2020; Kato et al., 2017). The inflammatory milieu, through TNF-α and IL-6 signaling, can stabilize MDM2 and synergize with p53-driven immunomodulation (Zhang et al., 2025). Beyond immune regulation, MDM2 influences mitochondrial metabolism via p53-independent mechanisms, creating vulnerabilities that can be exploited through combinations with PI3K or MAPK pathway inhibitors (Sun et al., 2024). In addition, MDM2 blockade in dendritic cells enhances maturation and antigen presentation, thereby boosting T-cell priming (Brummer and Zeiser, 2024). Collectively, these multifaceted effects highlight that MDM2 inhibition simultaneously alters tumor-intrinsic pathways and the broader immune landscape, reinforcing the rationale for carefully designed combination therapies that exploit p53-mediated immunity while mitigating resistance mechanisms.

The clinical translation of MDM2 inhibitors illustrates both the promise and perils of targeting fundamental cancer pathways. While the therapeutic rationale remains compelling, particularly for MDM2-amplified cancers, the narrow therapeutic window has prevented regulatory approval to date. Future success will require innovative approaches to maximize tumor-specific targeting while sparing normal tissues. Strategies include the development of PROTAC degraders with improved selectivity, nanoparticle-based delivery systems to enhance tumor drug concentration, and rational combination therapies that exploit synthetic lethality or protect normal tissues. Additionally, a better understanding of p53-independent MDM2 functions may identify contexts where lower doses can provide clinical benefit without dose-limiting toxicities. As these strategies mature, MDM2 inhibition may yet fulfill its potential as a transformative cancer therapy, but only through careful attention to the biological contexts that determine its therapeutic index.

## 5 Discussion and future directions

Targeting key molecular drivers such as RAS, PCNA, and MDM2 has revolutionized cancer therapeutics, providing broad-spectrum treatment strategies that address fundamental oncogenic processes across multiple malignancies. The development of RAS-targeted therapies has demonstrated the feasibility of directly inhibiting previously “undruggable” targets, while emerging inhibitors against PCNA and MDM2 offer novel avenues for disrupting tumor proliferation, DNA repair, and apoptosis regulation. Despite these advances, challenges such as intrinsic and acquired resistance, tumor heterogeneity, and treatment-related toxicity continue to hinder the long-term efficacy and clinical applicability of these therapies.

Future research should prioritize overcoming resistance mechanisms through rational combination therapies, synthetic lethality approaches, and adaptive treatment strategies. Integrating advanced drug delivery systems, PROTACs, and RNA-based therapeutics may enhance drug efficacy and specificity while mitigating toxicity. The incorporation of biomarker-driven patient stratification, real-time molecular profiling, and AI-assisted drug discovery will further refine treatment paradigms, enabling more precise and durable therapeutic interventions. As the field continues to evolve, the convergence of next-generation small-molecule inhibitors, immunotherapy combinations, and multi-targeted strategies holds great promise for improving patient outcomes. Continued investment in mechanistic research, translational studies, and clinical optimization will be critical in advancing molecularly guided cancer therapies, ultimately leading to more effective, personalized, and durable treatment options across diverse malignancies.

As aforementioned, in the review, we have discussed three representative oncogenes for targeted cancer therapy, focusing on pan-cancer approaches. When comparing pan-cancer and specific cancer strategies, several key considerations emerge. First, the choice between these approaches often hinges on the prevalence and significance of the molecular target. For common, well-characterized alterations that drive tumorigenesis across multiple cancer types, pan-cancer strategies offer a promising route. In contrast, when a target is highly specific to a particular cancer type or when the tumor microenvironment plays a critical role in therapy response, a cancer-specific approach may be more appropriate.

Second, patient selection is crucial in both strategies. The success of pan-cancer therapies depends on the accurate identification of biomarkers that reliably predict response across diverse tumor types. Advances in next-generation sequencing and bioinformatics have greatly improved our ability to stratify patients, yet challenges remain in standardizing these methods across different clinical settings. Similarly, cancer-specific strategies rely on precise molecular diagnostics to guide therapy, emphasizing the need for robust, validated assays that can be integrated into routine clinical practice.

Looking to the future, the integration of pan-cancer and cancer-specific strategies may offer the best of both worlds. A hybrid approach, where broad molecular targets are initially identified and then refined based on tumor-specific factors, could enhance treatment efficacy and overcome the limitations inherent in each strategy. Technological innovations such as artificial intelligence, multi-omics integration, and advanced imaging techniques are likely to play a pivotal role in this evolution, providing deeper insights into tumor biology and enabling more precise therapeutic interventions.

Moreover, the development of combination therapies represents a promising avenue for future research. By simultaneously targeting multiple pathways or mechanisms, combination regimens may address the complexity of tumor heterogeneity and reduce the likelihood of resistance. This approach requires a nuanced understanding of the interplay between various molecular targets, underscoring the importance of continued research and clinical innovation.

## 6 Conclusion

In this review, three oncogenic pathways, RAS, PNCA, and MDM2 are taken as examples in develop cancer targeted therapy using pan-cancer approaches, with key findings be applicable to specific cancer strategies. Both pan-cancer and specific cancer strategies represent significant strides in the ongoing battle against cancer. Pan-cancer approaches offer the promise of broad applicability and streamlined drug development by targeting common molecular alterations across diverse tumor types. On the other hand, cancer-specific strategies provide a high degree of precision and personalization by tailoring treatments to the unique characteristics of individual tumors. While each approach has its inherent strengths and limitations, the future of cancer therapy likely lies in an integrated strategy that leverages the advantages of both paradigms. As our understanding of cancer biology deepens and technological innovations continue to advance, the convergence of these strategies holds the potential to transform patient care. Ultimately, the goal remains the same, which is to develop effective, personalized therapies that improve outcomes and enhance the quality of life for cancer patients worldwide.
